# Whole-genome sequence diversity and association analysis of 198 soybean accessions in mini-core collections

**DOI:** 10.1093/dnares/dsaa032

**Published:** 2021-01-25

**Authors:** Hiromi Kajiya-Kanegae, Hideki Nagasaki, Akito Kaga, Ko Hirano, Eri Ogiso-Tanaka, Makoto Matsuoka, Motoyuki Ishimori, Masao Ishimoto, Masatsugu Hashiguchi, Hidenori Tanaka, Ryo Akashi, Sachiko Isobe, Hiroyoshi Iwata

**Affiliations:** 1 Department of Agricultural and Environmental Biology, Graduate School of Agricultural and Life Sciences, The University of Tokyo, Tokyo 113-8657, Japan; 2 Kazusa DNA Research Institute, Kisarazu, Chiba 292-0818, Japan; 3 Institute of Crop Science, National Agriculture and Food Research Organization (NARO), Tsukuba, Ibaraki 305-8518, Japan; 4 Bioscience and Biotechnology Center, Nagoya University, Nagoya, Aichi 464-8601, Japan; 5 Faculty of Agriculture, University of Miyazaki, Miyazaki 889-2192, Japan

**Keywords:** *Glycine max*, soybean, genome diversity, next-generation sequencing

## Abstract

We performed whole-genome Illumina resequencing of 198 accessions to examine the genetic diversity and facilitate the use of soybean genetic resources and identified 10 million single nucleotide polymorphisms and 2.8 million small indels. Furthermore, PacBio resequencing of 10 accessions was performed, and a total of 2,033 structure variants were identified. Genetic diversity and structure analysis congregated the 198 accessions into three subgroups (Primitive, World, and Japan) and showed the possibility of a long and relatively isolated history of cultivated soybean in Japan. Additionally, the skewed regional distribution of variants in the genome, such as higher structural variations on the *R* gene clusters in the Japan group, suggested the possibility of selective sweeps during domestication or breeding. A genome-wide association study identified both known and novel causal variants on the genes controlling the flowering period. Novel candidate causal variants were also found on genes related to the seed coat colour by aligning together with Illumina and PacBio reads. The genomic sequences and variants obtained in this study have immense potential to provide information for soybean breeding and genetic studies that may uncover novel alleles or genes involved in agronomically important traits.

## 1. Introduction

There are more than 1,750 gene banks existing in the world that store genetic resources comprised of 7.4 million accessions.^1^ However, <1% of these accessions have been used in breeding.^2^ Underutilization of genetic resources is partly due to the accessions being characterized insufficiently. Recent advances in DNA genotyping and sequencing technologies have enabled molecular descriptions for these accessions with genome-wide markers and whole-genome sequences. In rice, whole-genome sequence data of more than 3,000 accession genetic resources were collected and analysed.^3–5^ In soybeans, which is the species being investigated in the present study, 42,509 single nucleotide polymorphisms (SNPs) were determined from 20,087 accessions of genetic resources.^6^ Genomic information on genetic resources will enhance the utilization of genetic resources in plant breeding. A genome-wide association study (GWAS) facilitates the identification of genes/alleles, which can be utilized in plant breeding, from genetic variations within a germplasm collection.^7^ Genomic selection is a method of predicting the genetic ability of individuals and selecting individuals based on the prediction,^8^ which allows useful accessions to be identified from a germplasm collection.^9–11^

Whole-genome sequences collected from genetic resources provide useful information for plant breeding that can provide important clues and insights into the evolution and domestication process of crop species, subspecies, and cultivars.^4^ Whole-genome sequences also provide information that can determine genes and alleles that played important roles in local adaptation and artificial selection in the history of plant breeding.^12^ In addition, whole-genome sequences are expected to improve the power and precision of GWAS because they preserve rare variants and variants that are not in linkage disequilibrium (LD) with a reduced set of SNPs, such as SNPs genotyped with a genotyping array or a reduced‐representation sequencing approach. Genome structure variants, including copy number variations (CNVs), are also identified by whole-genome sequences.^13^ CNVs are known to have contributed to domestication and natural or artificial selection in the history of plant breeding and has been linked to important agronomic traits.^13^^,^^14^ Moreover, whole-genome sequences of genetic resources can also be useful as a reference panel to impute whole-genome polymorphisms in large experimental/breeding populations genotyped with a reduced set of SNPs to suppress the time and cost of genotyping.^15–17^ From the above-mentioned various viewpoints, studies on whole-genome sequences of genetic resources have been performed in various crop species (e.g. rice,^3–5^ maize,^18^ sorghum,^19^ tomato,^20^ and soybean^12^).

Globally, soybean [*Glycine max* (L.) Merr.] is the most important legume and is also the fourth in worldwide production after rice, wheat, and maize in terms of global crop production. The soybean is an important source of protein, an oil crop, and is used for both food and animal feed. Recently, numerous functional constituents to human health found in soybean seeds have attracted attention,^21^ and it may also be used as a biofuel crop.^22^ In addition, the crop can also provide nutrients to the soil through nitrogen fixation. Because of these characteristics, the importance of soybean as a crop has been increasing in recent years. Hence, a wide variety of genetic variants has been collected as genetic resource collections for soybeans, including its wild species, *G. soja* (Sieb. & Zucc.) as genetic resources for breeding.

After the genome sequence of soybean became available,^23^ the whole-genome sequences from soybean genetic resources were collected and used for studies in domestication and varietal improvement of soybean.^12^^,^^24–29^ A relatively high rate (55,^24^ 45,^25^ 60,^26^ 21,^12^ and 7%^27^) of wild soybean (*G. soja*) accessions were included in the materials in the previous whole-genome sequencing studies in soybean, and the number of *G. max* accessions was <100, except for Zhou et al.^12^ In the Zhou et al.^12^ study, they resequenced 302 accessions, including 170 accessions (86 landraces and 84 improved cultivars) of *G. max*, and analysed genomic variation dynamics during the domestication and varietal improvement of soybean. The number of accessions used in the study was much larger than that in earlier studies, and this may have contributed to the successful detection of traces of selection and variants associated with domestication and improvement traits. Although the genetic resource with the whole-genome sequences employed in Zhou et al.^12^ is useful for future soybean breeding, we consider that the increase in the number of accessions with whole-genome sequences contribute to improving the statistical power for detecting traces of selection and the power of GWAS.^30^

Soybean is assumed to have been domesticated in China around the eleventh century BC and then disseminated to surrounding countries around the first century A.D.^31^ Accordingly, the genetic and morphological characteristics of Japanese soybean are distinct from those grown across the Asian continent.^32^^,^^33^ In Japan, various soybeans have been used to prepare traditional foods such as tofu, fermented beans, miso, boiled beans, soy sauce, and vegetable beans. Because the seed size of Japanese soybeans is one of the important characteristics of these foods, range of the variation is approximately 2-fold greater than the rest of the world’s soybeans.^33^ In addition, colour traits, especially the seed coat and hilum colour, are important features for food processing such as the yellow seed coat with a pale hilum colour for tofu production, black or green seed colour for boiled beans, and the non-yellow colouring or stay-green characteristic of the pod for vegetable beans. The genetics of these colouration traits and their respective genes have been well characterized. As for the green-colour related traits, homozygosity of two recessive alleles at classical loci, *D1* and *D2*, or a cytoplasmic gene, *cytG*, are known to cause the stay-green phenotype of the pod and seed coat at maturity.^34^ The recessive *d1* and *d2* alleles have a mutation that causes the loss of function in the Stay‐Green (SGR) proteins,^35^ while *cytG* has an insertion that causes a frameshift in the chloroplast *psbM* gene.^36^ The *G* allele at the *G* locus produces green-coloured seeds using different mechanisms from these loci,^37^ and is dominant over the yellow seed. The *G* allele encodes a CAAX amino-terminal protease protein, while the *g* allele has a mutation that causes the loss of the last transmembrane domain.^38^

Epistasis among classical colour loci, such as *I*, *R*, *T*, and *W1* have been reported to occur because the compounds responsible for the colouration of the seed coat, hilum, pubescence, and flower in soybean are mostly related to the biosynthetic pathway of anthocyanins.^39^ The dominant *I* allele inhibits seed coat pigmentation and causes a uniformly yellow seed coat and hilum, whereas the *i* allele allows pigmentation. The other *i^i^* and *i^k^* restrict pigmentation to the hilum and to the saddle-shaped region around hilum, respectively. The mechanisms for *I* and *i^i^* alleles have been characterized as RNA silencing of chalcone synthase (*CHS*) genes in the biosynthetic pathway of anthocyanins.^40^^,^^41^ With the combination of *i* allele, the *R* and *r* alleles at the *R* locus result in a black and brown hilum/seed coat, respectively. The *R* gene encodes the R2R3 MYB transcription factor, which might control the expression of UDP-glucose: flavonoid 3-O-glucosyltransferase (UF3GT) in the final step of anthocyanin biosynthesis, and the *r* allele is caused by four types of loss-of-function mutations.^42^ The *T* and *W1* loci are known to control pubescence and flower colour, respectively, in addition to epistatic interactions for the pigmentation of seed colour traits. The dominant *T* allele produces tawny pubescence, whereas the recessive *t* allele with a single-base deletion causes a loss of function in flavonoid 3′-hydroxylase and produces grey pubescence.^43^ The dominant *W1* allele produces a purple flower and hypocotyl phenotype, while the recessive *w1* allele with a single-base deletion causes a loss of function in flavonoid 3′,5′-hydroxylase and produces a white flower and green hypocotyl phenotype.^44^

Understanding the genetic control of flowering time and maturity is indispensable to efficiently develop a new variety with a photoperiodic adaptation to different latitudes. For that reason, the genes responsible for *E1*,^45^*E2*,^46^*E3*,^47^*E4*,^48^*E9*,^49^ and *J*^50^ were isolated among the 10 major classical loci (*E1*–*E9* and *J*). In addition, genes for other loci such as *qDTF*-J,^51^*E1-like-b*,^52^ and *GmPRR3*^53^ were isolated. The next important task would be to accumulate allelic information from the breeding materials since different allelic combinations of these loci determine adaptations to a different latitude. However, functional redundancy between duplicated gene copies in the soybean genome makes it difficult to understand the relationship between genetic variation and agronomically important traits. Therefore, a resequencing effort for many accessions will provide a chance to comprehensively identify new alleles and genes that potentially affect agronomically important traits apart from flowering time and maturity.

In this study, we collected and analysed the whole-genome sequences of 198 soybean accessions. The accessions were mainly from two soybean mini-core collections from the National Agricultural and Food Research Organization (NARO) Genebank. The accessions in the mini-core collections were carefully selected from 1,603 accessions,^33^ based on the polymorphisms of 191 SNP markers and several agronomic traits, to ensure that the collections retained as many genetic variations as possible in all accessions. In this study, we characterized the polymorphisms found in the whole-genome sequences and investigated the subpopulation structure and levels of genetic differentiation in the accessions based on their polymorphisms. In addition, using the number of days to flowering (DTF) as an example trait, we evaluated the potential of GWAS using whole-genome sequences. Among the 198 accessions, we also employed 10 for long-read sequencing to analyse large structural variants. Copy number variants were also identified using Illumina reads to investigate the genome structure variance. The variations in colour related to the seed coat, hilum, pubescence, and flower were characterized as polymorphisms in the whole-genome sequences, and their relations with known genes were investigated. Through these analyses, we evaluated the potential significance of whole-genome sequences being prepared for soybean genetic resources. The whole-genome sequences collected for the genetic resources will facilitate the active use of genetic resources in soybean breeding programs.

## 2. Materials and methods

### 2.1. Plant materials

In this study, we utilized 198 soybean accessions for whole-genome sequencing (Supplementary Table S1): 192 accessions from Japanese and world soybean mini-core collections,^33^ an Indian cultivar ‘L323’ (JP241838), and a Japanese cultivar ‘Misuzudaizu’ (JP28856) obtained from NARO Genebank (https://www.gene.affrc.go.jp/index_en.php, 15 January 2021, date last accessed); Japanese landrace ‘Houjaku Kuwazu’ (PI416937) and a United States (US) cultivar ‘5002T’ (PI634193) obtained from the USDA (United States Department of Agriculture) germplasm collection through GRIN (Germplasm Resources Information Network). A soybean cultivar ‘Norin2’ and a *Glycine soja* accession (B01167) were obtained from the National BioResource Project (https://www.legumebase.brc.miyazaki-u.ac.jp, 15 January 2021, date last accessed).

Two plants were grown with an inter-row spacing of 80 cm and a hill spacing of 20 cm in the field at NARO in Tsukuba, Ibaraki, Japan (36°01′25.6″ N 140°06′59.1″E). Seeds were sown on June 1, 2010, and the DTF of 184 successfully germinated accessions were recorded for association analysis. Of the 198 accessions, 14 were excluded from the evaluation of DTF because six and eight accessions had not planted in the field and germinated late due to overseed, respectively. The colour of hypocotyl, flower, pubescence, leaf at maturity, and seeds of these plants were recorded while comparing that of the soybean reference cultivar Williams 82 (accession no. GmWMC115 in the present study) with yellow seed with black hilum, tawny pubescence, and white flowers (*i^i^*, *T*, *R*, *w1*).^54^

### 2.2. Illumina whole-genome sequencing

Freeze-dried young leaves collected from a plant in each accession were ground using a mortar and pestle. Total DNA was extracted from the finely ground leaf tissue using the DNeasy Plant Mini Kit (Qiagen, Hilden, Germany). The DNA was physically sheared into ∼350 bp fragments using Covaris S2 (Covaris, Brighton, UK). The fragmented DNA was used for DNA library construction with the TrueSeq DNA PCR-Free Library Prep Kit (Illumina, San Diego, CA). The DNA libraries were sequenced using the Illumina HiSeq X Ten or HiSeq 4000 (Illumina).

### 2.3. PacBio whole-genome sequencing

PacBio whole-genome sequencing was performed for 10 accessions: ‘Misuzudaizu’ (JP28856), ‘Enrei’ (GmJMC025), ‘Houjaku Kuwazu’ (PI416937), ‘Fukuyutaka’ (GmJMC112), ‘Moshidou Gong 503’ (GmWMC084), ‘Peking’ (GmWMC084), ‘PK 73-54’ (GmWMC071), ‘L323’ (JP241838), ‘5002T’ (PI634193), and ‘Williams 82’ (GmWMC115; Supplementary Table S1). For PacBio sequencing, the total DNA was extracted from finely ground leaf tissue using an SDS-based DNA extraction method^55^ and was used for SMRTbell libraries (Pacific Biosciences, Menlo Park, CA). Sequences were generated using PacBio Sequel (Pacific Biosciences).

### 2.4. Reference genome sequences and annotation data used in this study

Gmax_275_v2.0 softmasked sequences and the genome annotation of Williams 82, which was a completely sequenced soybean accession,^23^ were used as the reference for the analyses of this study. The reference data were obtained from Phytozome 12.1,^56^ and the gene annotations and IDs described in this article were also based on the descriptions in Phytozome.

### 2.5. Variant call and diversity analysis using Illumina reads

The Illumina reads were trimmed with Trimmomatic version 0.36^57^ with the following parameters: ‘ILLUMINACLIP: TruSeq3-PE-2.fa: 2:30:10 LEADING: 3 TRAILING: 3 SLIDINGWINDOW: 4:15 MINLEN: 36’. The trimmed reads were mapped on the reference sequence using the BWA-aln (release 0.7.17) algorithm with default options.^58^ The mapped reads were then sorted using SAMtools release 1.7,^59^ and duplicates were removed using Picard tools (release 2.18.3; http://broadinstitute.github.io/picard/, 15 January 2021, date last accessed).

The variants for each accession were called using the GATK HaplotypeCaller (release 4.0.4.0) with the ‘.g.vcf’ extension.^60^ GATK GenomicsDBImport and GenotypeGVCFs were used for joint genotyping to produce a single VCF per sample of GVCF. Then, variants underwent quality assessment using the GATK best practices pipeline (https://software.broadinstitute.org/gatk/best-practices/, 15 January 2021, date last accessed) to obtain a raw VCF that passed through the variant filtration step. The initial step for the variant dataset contained 10,116,707 SNPs and 2,835,680 indels. Detailed methods for the preparation of the variant data are described in Supplementary Methods and Fig. S1.

### 2.6. Genetic and genomic diversity analyses

The genetic structure of the population was estimated using phylogenetic analysis, principal component analysis (PCA), and ADMIXTURE^61^ analysis based on whole-genome sequences. For the phylogenetic analysis, we constructed a neighbour-joining (NJ) tree based on the whole-genome genetic distances among accessions, calculated with the Jukes and Cantor model^62^ using the ape package^63^ in R.^64^ In constructing the NJ tree, the accession ‘B01167’, which is the only accession of *G. soja*, was treated as an outgroup. For PCA, we calculated whole-genome Euclidean distances among the accessions based on their genotypes and performed multi-dimensional scaling based on the distances using the ‘cmdscale’ function in R. We performed ADMIXTURE analysis^61^ with the models of one to eight subpopulations to estimate the ancestries of the accessions. A 5-fold cross-validation was performed for each number of the subpopulations to select the appropriate K value.

We calculated nucleotide diversity (*π*), pairwise and total *F_ST_*, and *r^2^* to measure the LD for the entire genome with non-overlapping 500 kb windows, and to evaluate genome-wide pattern levels of polymorphisms, genetic differentiation, and LD. For this calculation, we used an in-house developed R program. To identify CNVs among the 198 soybean accessions, CNV-Seq with the last updated version in 2014^65^ was performed based on the Illumina reads with the option of 100 kb window-size. The Illumina Williams 82 reads were used as reference reads.

### 2.7. GWAS of flowering date and seed weight

We performed GWAS for the flowering date to demonstrate the viability of the identified variants. The numbers of days from sowing to first flowering of 184 accessions were used for the association analysis. SNPs with minor allele frequencies (2.5%) or whose missing rate was more than 5% were filtered out for the GWAS study. Imputation was conducted using Beagle 5.0 with default parameter settings.^9^ GWAS was performed using a linear mixed model^60^ implemented by the ‘association.test’ function in gaston package ver. 1.5.5^60^ in R. In the linear mixed model, the first two principal components of marker scores were included as fixed effects. A genetic relationship matrix specifying a random additive effect was computed using the ‘GRM’ function of the gaston package. The *P* values of the marker-trait associations were calculated using the Wald test. The genome-wide significant threshold was obtained based on a false discovery rate (FDR^66^) at a 1% level. Manhattan plot of GWAS was created using qqman^67^ and CMplot package (https://github.com/YinLiLin/R-CMplot, 15 January 2021, date last accessed) in R.

### 2.8. Structural variation analysis

Structural variation (SV) detection was performed using PacBio reads from the 10 soybean accessions. The PacBio reads of each accession were mapped onto the reference genome sequence using NGMLR^68^ version 0.2.6. SV detection from mapped results was performed using SAMtools version 1.3.1^59^ and Sniffles version 1.0.8.^68^ Genome-wide distribution of SVs (insertions, deletions, and duplications) was filtered by length, where the value of SVLEN in VCF file ≥1 kb and ≤50 kb, and grouped into three categories: Japan, Primitive, and World, which were performed using in-house Perl scripts. The integrated genome maps were then illustrated by CIRCOS 69-3.^69^

### 2.9. Identifying the variation in PacBio and Illumina reads related to the I locus

The presence and absence of variation (PAV) related to the *I* locus were analysed based on the mapping results of PacBio and Illumina reads onto the reference genome. In addition, the genomic positions of the *CHS* genes of BAC77G7-a and BAC56G2 (GenBank accession numbers: EF623854 and EF623856); which were reported as BAC clones that covered the *I* locus of Williams 82,^70^ were compared with Gmax_275_v2.0 using MUMmer3.^71^ PAV of *Gm-c1069-6017*^41^ and *GmICHS*^40^ of the *I* locus and *GmD2IN*^35^ of the *D1* locus were manually identified by comparing the alignments using IGVtools 2.4.11^72^ and CLC Genomics Workbench 12 (Qiagen, Hilden, Germany).

## 3. Results and discussion

### 3.1. Whole-genome sequencing of the soybean accessions

A total of 25 billion paired-end Illumina reads were obtained for the 197 *G. max* and one *G. soja* accessions (Supplementary Tables S1 and S2). The mean depth of the reads against the soybean genome ranged from ×6.8 to ×32.9 with an average of ×16.3. The mapping ratio onto the ‘Williams 82’ reference was 94.7% on average, and the mean covered ratio on the reference genome was 93.6%. A total of 12,952,387 variants, including 10,116,707 SNPs and 2,835,680 insertions/deletions (indels), were identified as the results of the variant call and filtering (Table 1). The information for variants among accessions can be compared using the multiple genome Browser TASUKE^73^ from https://daizutasuke275-core.daizu.dna.affrc.go.jp/ (15 January 2021, date last accessed).

**Table 1:** Number of variants identified on the 198 soybean accessions

Chr	Raw SNP	Raw INDEL	Total raw variant	Filtered SNP	Filtered INDEL	Total filtered variant
Chr01	809,046	139,387	948,433	519,110	133,213	652,323
Chr02	685,281	138,448	823,729	464,550	133,805	598,355
Chr03	918,813	165,558	1,084,371	581,996	160,415	742,411
Chr04	858,671	147,249	1,005,920	564,337	141,912	706,249
Chr05	560,582	110,072	670,654	361,072	105,493	466,565
Chr06	925,223	171,679	1,096,902	579,010	166,158	745,168
Chr07	712,969	143,049	856,018	463,300	138,337	601,637
Chr08	693,324	147,969	841,293	456,798	143,504	600,302
Chr09	743,949	141,684	885,633	481,385	136,143	617,528
Chr10	714,099	134,919	849,018	462,054	129,213	591,267
Chr11	438,702	95,978	534,680	303,411	92,734	396,145
Chr12	595,004	115,856	710,860	391,525	111,748	503,273
Chr13	722,750	160,407	883,157	481,921	156,154	638,075
Chr14	902,138	151,259	1,053,397	590,557	145,723	736,280
Chr15	1,048,862	175,748	1,224,610	648,774	170,120	818,894
Chr16	861,304	165,506	1,026,810	526,504	160,445	686,949
Chr17	642,407	125,991	768,398	425,846	122,022	547,868
Chr18	1,272,177	224,567	1,496,744	794,961	217,759	1,012,720
Chr19	804,396	144,788	949,184	512,977	139,773	652,750
Chr20	767,005	136,478	903,483	506,619	131,009	637,628
Total	15,676,702	2,936,592	18,613,294	10,116,707	2,835,680	12,952,387

### 3.2. Genetic diversity and population structure analyses

**Figure 1:**
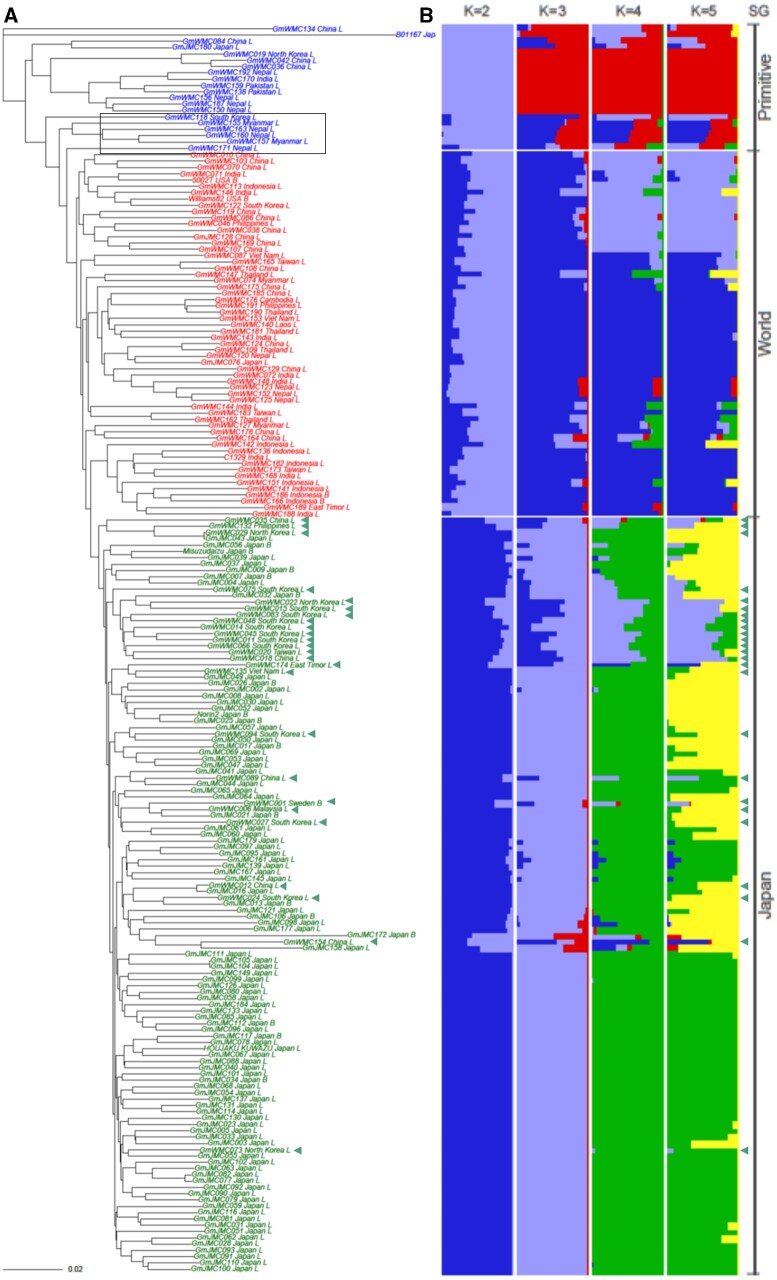
The phylogenetic relationships and Population structure of 198 soybean accessions. (A) A neighbour-joining dendrogram phylogenetic tree of the 198 soybean accessions. The accessions were classified into three subgroups: ‘Primitive’, ‘World’, and ‘Japan’. (B) Population genetic structure estimated by Admixture analysis. Results from *k* = 2 to 5 are shown. Green triangles indicate the non-Japanese accessions in the ‘Japan’ subgroup.

An NJ tree based on whole-genome SNPs was built to investigate the phylogenetic relationships among the 198 accessions (Fig. 1A). The NJ tree indicated that the 198 accessions were clustered into three subgroups, two of which were not monophyletic. Based on the origins of accessions consistent with each subgroup, we named the subgroups as ‘Primitive’, ‘World’, and ‘Japan’. The ‘Primitive’ subgroup was comprised of the *G. soja* accession (B01167), ‘Peking’ (GmWMC084), ‘Moshidou Gong 503’ (GmWMC042), and 17 other accessions (Supplementary Table S1). The World subgroup consisted of ‘Williams 82’ (GmWMC115), ‘5002T’, ‘PK 73-54’ (GmWMC071), and 55 other accessions. The ‘Japan’ subgroup consisted of a monophyletic cluster of 120 accessions, which were mainly Japanese and Korean landraces/cultivars.

PCA showed a similar diversity pattern to the NJ tree (Supplementary Fig. S2A). In PC1, which accounted for 9.0% of the total variations, the accessions were divided into three subgroups in the order of ‘Primitive’, ‘World’, and ‘Japan’. PC2, which accounted for 3.4% of the total variations, mainly captured the genetic differentiation between ‘Primitive’ and ‘World’, compared to ‘Japan’, which presented intermediate scores between the two subgroups. In both PC1 and PC2, the distribution of the three subgroups was not completely discrete, indicating that the genetic differentiation between these subgroups is continuous and was likely due to the migration between subgroups. Landraces and breeding lines were the two types of accessions in the ‘World’ and ‘Japan’ subgroups. However, there were no clear differences between the distribution patterns of breeding lines and landraces in both ‘World’ and ‘Japan’ subgroups. In PC3 and PC4, no clear patterns were observed among the subgroups (Supplementary Fig. S2B), suggesting that the PC1 and PC2 scores are useful indicators for determining which accession belongs to each subpopulation.

The admixture analysis also showed a similar diversity pattern with the NJ tree and PCA (Fig. 1B). Each of the three subgroups showed a similar genetic background among accessions when *K* = 3. Some accessions (e.g. six accessions of ‘Primitive’ which are in the grey box in Fig. 1A) had intermediate genetic backgrounds between subgroups (e.g. the six accessions had intermediate backgrounds between ‘Primitive’ and ‘World’). Moreover, the cross-validation error was the smallest when *K* = 5 (Supplementary Fig. S3), suggesting that a significant subpopulation structure may exist even within each of the three subgroups. Thus, the 198 accessions did not fall into three distinct subpopulations and had a stratified and complex structure among them. Nevertheless, the two boundaries between the three subgroups (white dashed lines in Fig. 1B) were visually identifiable, and thus the classification into the three subgroups was considered reasonable, even based on the result of Admixture analysis.

The 12,953,387 variants were classified based on their shared degree, and approximately one-third (4,486,367 variants) were commonly observed among the three groups. These variants were regarded as the ancient variants rather than ‘private’ variants, which identified single groups only. The private variants per subgroup were defined as variants that had been observed only in a specific group. The number of private variants per subgroup was higher in ‘Japan’ (2,299,690) than in ‘World’ (1,119,074) or ‘Primitive’ (2,145,920; Supplementary Fig. S4). These private variants were considered as candidate causal variants that ascribe group-specific phenotypes. The allele frequencies of variants in each group (subpopulations and shared categories) were calculated to elucidate genetic diversity. The average, median, and mode of allele frequencies were higher in ‘Primitive’ than in ‘World’ or ‘Japan’ (Supplementary Table S3).

The functional impact of the 12,953,387 variants (10,117,707 SNPs + 2,835,680 indels) was estimated using SnpEff (Supplementary Table S3 and Fig. S5A) to investigate and exploit genotypic and phenotypic variations. Out of all variants, 29,655,355 (97.45%) were classified into ‘Modifier’ (intergenic variants), while 9,757 (0.24%) were classified into ‘High’ (frameshift variant, stop lost/gained), 358,102 (1.26%) into ‘Moderate’ (coding sequence variant), and 319,839 (1.05%) into ‘Low’ (synonymous variant). The number of private variants classified as ‘High’ was more than twice (20,283) in ‘Japan’ than those in ‘Primitive’ (9,540) and ‘World’ (8,993) (Supplemental Table S3). For the functional classification of SnpEff, 613,799 variants were classified as ‘Nonsense’ (9,757; 1.6%), ‘Missense’ (358,102; 58%), and ‘Silent’ (245,940; 40%) as shown in Supplementary Table S3 and Fig. S5B. The ‘Japan’ subgroup had the largest number of private variants classified into ‘Nonsense’ and ‘Missense’ among the three subgroups (Supplementary Fig. S5B). These variants may implicate phenotypic variations among populations.

We evaluated the genome-wide diversity in the whole-genome sequences by calculating the nucleotide diversity (π), genetic differentiation (pairwise and total $F_{ST}$), and linkage disequilibrium ($r^{2}$) in 500 kb non-overlapping windows for each of the three subgroups and all subgroups together. As a result, the nucleotide diversity was highest in ‘Primitive’ and lowest in ‘Japan’ in most genomic regions (Supplementary Fig. S6A). The private variants, defined as variants that had been observed only in a specific accession, were counted in each accession. The average number of private variants per accession was higher in ‘Primitive’ (62,329) than in ‘World’ (7,091) or ‘Japan’ (6,584). The ‘Japan’ subgroup had the largest number of accessions (120 of 198), so it is reasonable for it to have a larger number of private variants per subgroup. The genetic differentiation between subpopulations was generally largest in the comparison between ‘Primitive’ and ‘Japan’ and lowest in the comparison between ‘World’ and ‘Japan’ in most of the genomic regions (Supplementary Fig. S6B). Some genomic regions, however, showed different patterns from the general pattern; for example, the differentiation in the terminals of the long arms of chromosomes 3 and 5 was the lowest between ‘Primitive’ and ‘World’, while it was high between ‘Primitive’ and ‘Japan’ and ‘World’ and ‘Japan’. The LD was the highest in ‘Primitive’ and the lowest in ‘Japan’ (Supplementary Fig. S6C), and studies have reported that small populations have higher LD value than large populations.^74^ As in the case of genetic differentiation, some genomic regions showed different patterns from the general pattern in LD. For example, we only observed the peak of the LD in the middle (∼25 Mb) of the chromosome 6 in ‘Japan’, while the peak of the LD in the middle (∼10 Mb) of the chromosome 7 was in ‘World’.

### 3.3. GWAS for flowering date

**Figure 2:**
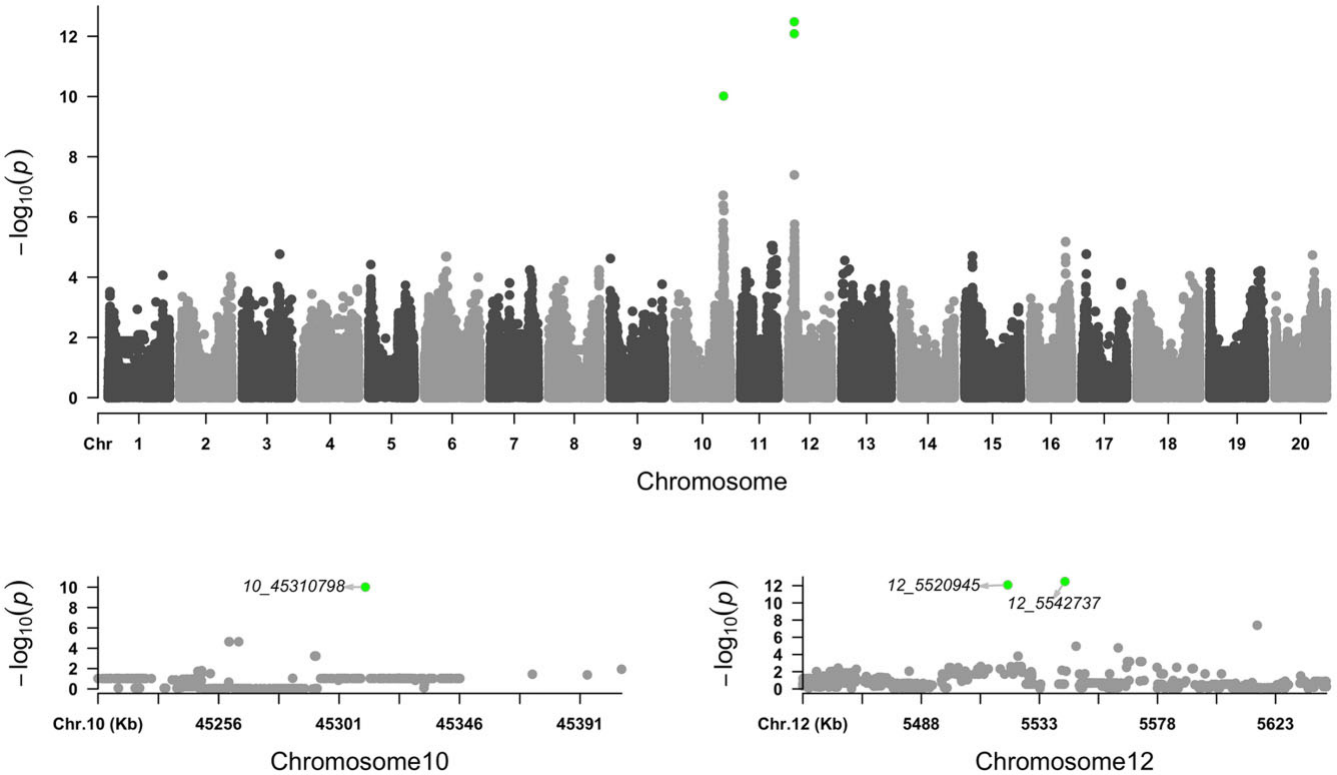
Manhattan plots of GWAS for flowering time in 2010. Significantly positive SNPs (FDR <0.01) are highlighted in green. (A) Whole-genome, (B) chromosome 10, and (C) chromosome 12.

A genome-wide association test was performed on DTF with 4,776,813 genome-wide SNPs (Fig. 2) to demonstrate the strength of whole-genome GWAS with mini-core collections. With a 1% threshold of FDR, three significant associations were detected at the 5,520,945 and 5,542,737 bp positions on chromosome 12, and at the 45,310,798 bp position on chromosome 10. The variants of *e2*, *e3-tr*, and the stop-loss variant (rs125308117) of *two-component response regulator*-like gene on Chr12 were significant in a gene-based association test for flowering time in the mini-core collection. Ogiso-Tanaka et al.^75^ estimated a large deletion on E3 by the coverage of four amplicons on the 4th exon. In this study, *e3-tr* could not be incorporated into the association analysis due to the difficulty of detecting a 15 kb deletion in *E3* using whole-genome sequences.

**Figure 3:**
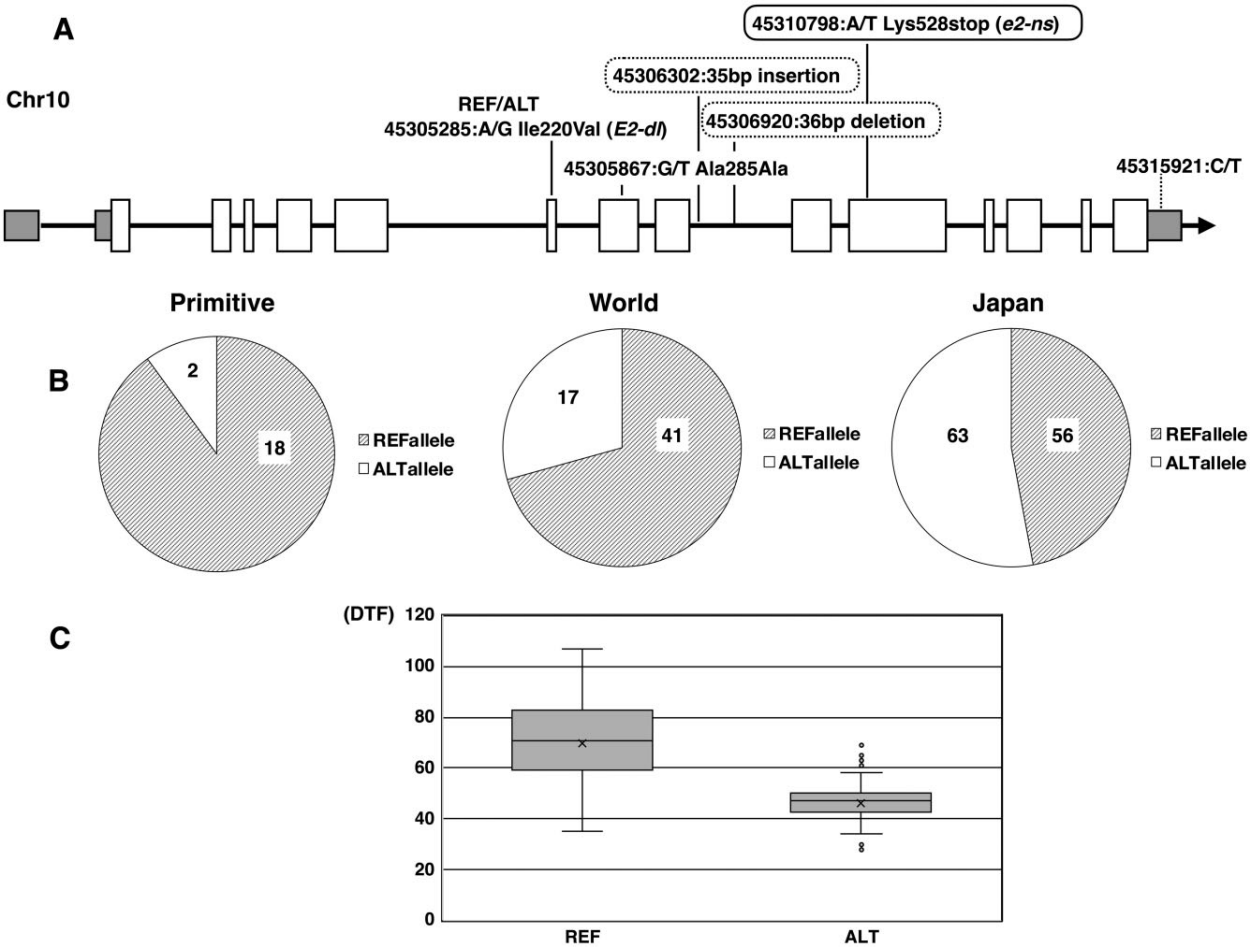
Sequence variants on the *GIGANTEA* gene (Glyma.10G221500), the allele frequency of a targeted SNP, and distribution of flowering date. (A) The structures of Glyma.10G221500 encodes the soybean *GIGANTEA* gene and identified variants. Black arrow—gene direction, white box—coding exon, and UTR—grey box. Genomic positions represent variants identified by Illumina reads in the 198 soybean accessions. Those enclosed with solid and dotted lines were basic variants used in GWAS and SVs detected by PacBio, respectively. (B) Allele frequency in the ‘Primitive’, ‘World’, and ‘Japan’ subgroups of the 198 accessions of the SNP (Ch10-45310798) that showed the highest significance on the gene by the GWAS analysis. Ref (same as the reference sequence, Williams 82) and Alt alleles are ‘A’ and ‘T’, respectively. The numbers in the pie charts indicate that of accessions having the corresponding homozygous alleles. One accession in the ‘Japan’ subgroup showed hetero allele and exclude from the chart. (C) Boxplots of flowering date in the 184 accessions having Ref (left) and Alt (right) allele on the SNP (Chr10, 45310798). The vertical line indicates the days to flowering (DTF).

The significant association detected in the SNP at 45,310,798 bp on chromosome 10 was in the Glyma.10G221500 coding region, which encodes the classical *E2* gene, that is, a soybean *GIGANTEA* gene.^44^ The alleles of the SNP were ref (same as the reference sequence, ‘Williams 82’) ‘A’ and alt ’T’ alleles, which corresponded to Lys (AAA) and a premature stop codon (TAA) at the 528 amino acid sequence position, respectively (Fig. 3A). The proportion of the alleles was different among the subgroups, especially when comparing ‘Primitive’ to ‘World’ and ‘Japan’ (Fig. 3B). Based on the relationships between DTF phenotypes and SNP alleles, the reference (ref: A) and alternative (alt: T) alleles were associated with late and early flowering phenotypes, respectively (Fig. 3C). Previously, three alleles, *E2-in* (Williams 82), *E2-dl*, and *e2-ns*, have been identified from 63 accessions covering several ecological types by sequencing of the genomic region of the *E2* locus.^76^ Wang et al.^77^ identified 47 haplotypes of *GIGANTEA* from 233 Chinese soybean and 104 wild soybean accessions. Among them, three amino acid sequence haplotypes, H1 (*e2-ns)*, H2 (*E2-dl*), and H3 (*E2-in*), have been reported to be in the cultivated soybean gene pool. Interestingly, five novel variants with amino acid changes were obtained in the present study (Supplementary Table S4). It is necessary to confirm whether these novel variants, especially the novel nonsense variant of Gln53stop in GmJMC041 and GmJMC044 that has no Lys528stop mutation, affect the flowering time or maturity and can be called a new allele.

The SNP at 5,520,945 bp on chromosome 12 showed a significant association in the coding region of Glyma.12G073900, which encodes the clock-associated pseudo-response regulator 3 (*GmPRR3b*).^53^ This SNP, which is located at the first nucleotide of the termination codon ‘TAA’, changes the codon to ‘CAA’ of Gln on the alt allele. This caused a shift of the stop codon at the position of 627 aa in the ref allele (5,520,945 bp) to 795 aa in the alt allele (5,521,025 bp, Supplementary Fig. S7A). The proportion of the alleles was significantly different in ‘Primitive’ compared to ‘Japan’ and ‘World’ (Supplementary Fig. S7B). Accessions with the ref allele tended to flower early, while accessions with the alt allele tended to flower late (Supplementary Fig. S7C).

There are two landraces in the ‘Japan’ subgroup, ‘Kurodaizu’ and ‘Hiku Anda’ (GmJMC030 and GmJMC049), which originated from Okinawa prefecture located in the south most part of Japan. It was reported that these landraces belonged to a cluster that consisted of old cultivars known as the precocious summer-type soybean.^33^ We confirmed that the two landraces had early flowering alleles in the *E2* gene and *GmPRR3b*, supporting that the two landraces belonged in the early flowering group in the previous study.^33^ The ‘Japan’ subgroup also includes three landraces and three breeding lines, ‘Waseousode’ (GmJMC005), ‘Tokachi Nagaha’ (GmJMC007), ‘Shizunai Daizu’ (GmJMC009), ‘Ooyachi 2’ (GmJMC021), ‘Bansei Hikarikuro’ (GmJMC033), and ‘Yakumo Meaka’ (GmJMC037), that originated from Hokkaido which is the northernmost prefecture of Japan. These six accessions had early flowering alleles in both genes, suggesting an adaptation to specific environmental conditions in the northern part of Japan.^31^

Li et al.^78^ reported six variants of *GmPRR3b* that caused amino acid changes and eight haplotypes from 383 accessions, including wild soybean accessions. We were able to identify four novel variants causing amino acid changes in the current study (Supplementary Table S4). Among the eight haplotypes determined by Li et al.,^78^ H1 and H6 encoded truncated peptides. The H6 haplotype is the most frequent haplotype in cultivated soybeans, including the reference ‘Williams 82,’ and was frequently observed as a ref allele in our study, while H1 was found only in wild soybeans. The next would be the H4 and H5 haplotypes, which encode longer amino acid sequences, that were reported to flower later than H1 and H6. Similarly, H4 and H5 haplotypes were second and third most frequent haplotypes (33 and 19 accessions, respectively) in our study. The H7 and H8 haplotypes, which encode the same length of amino acid sequence with a Ser100Leu mutation in the pseudo receiver domain, have been reported to flower earlier than H4 and H5.^74^ The novel mutation of Asp98Asn, which was only found in haplotype H5, was located in the same pseudo receiver domain. Together with the other novel frameshift variant of Gly556fs in GmWMC087, which causes a shorter truncated protein than the H6 haplotype, further studies are necessary to understand the allelic effect of the novel variants on flowering time and maturity.

### 3.4. SVs between nine soybean accessions

PacBio reads were obtained for 10 accessions originating from Japan, China, India, and the USA (Fig. 1, Supplementary Table S1). The total length of the subreads ranged from 10.3 to 17.7 Gb, representing 10.5 to 18.1 × of the soybean genome. The average subread lengths ranged from 6,777 to 8,019 bp. SVs for the 10 soybean accessions with PacBio reads were identified and compared (Table 2). ‘Williams 82’, the accession of the reference genome, was identified to have a total of 2,033 SVs. The number represents the degree of errors (false positive or miss-assembly of the reference genome). Overall, the number of SVs with complex structures such as duplications was less than insertions and deletions. A large number (a total of 16,363) of insertion and deletion polymorphisms existed in two accessions in the ‘Primitive’ subgroup. The polymorphisms of these two accessions were newly detected by long-read resequencing, which was at a moderate genetic distance from the reference genome and will further contribute to novel genetic analysis. This result agrees with the result of the PCA analysis (Supplementary Fig. S2), which was based on single-nucleotide variants.

**Table 2:** The numbers of the SVs detected from 10 soybean accessions with PacBio reads

	Primitive		World				Japan			
	Peking	Moshidou Gong 503	C1329	PK 73-54	5002T	Williams 82	Houjaku Kuwazu	Enrei	Fukuyutaka	Misuzudaizu
Total	17,922	25,002	9,128	6,373	5,335	2,033	14,392	14,921	15,982	6,144
Insertions	7,388	10,959	3,878	2,645	2,151	785	6,254	6,432	6,764	2,486
Deletions	8,975	12,475	4,348	2,885	2,454	452	6,671	7,043	7,458	2,821
Duplications	370	356	156	166	126	132	356	370	348	143
Inversions	97	106	47	44	47	41	100	90	92	46
Translocations	1,092	1,106	698	632	557	623	1,011	985	1,315	648
Inverted duplications	0	0	1	1	0	0	0	1	5	0

Genome-wide distributions of SVs among the three groups are shown with π values in Supplementary Fig. S8. It has been reported that the distributions of SVs and SNPs tend to be positively correlated by LD analysis.^5^^,^^79^^,^^80^ However, several exceptions, such as on 10–20 Mb on Chr03 and 10–30 Mb on Chr19 in the ‘Japan’ subgroup, were observed. The fewer π values and higher SVs in these regions tend to be negatively correlated, suggesting that it is difficult to detect an associations between the phenotype and DNA polymorphisms using SNP-based analysis even if there is phenotypic diversification due to SVs in the regions.^81^ Furthermore, we focused on regions where genes related to domestication or breeding are located. For example, the regions at 37–41.5 Mb on Chr14 and 10–20 Mb on Chr20 showed lower polymorphisms in ‘World’ and ‘Japan’ than ‘Primitive’. According to Zhou et al.,^12^ these regions were considered as putative selective sweep regions of seed oil contents (Chr14: 37,550,001.41,300,000, Chr20: 10,270,001.18,460,000) caused by the breeding or domestication process.^12^ The lower polymorphic regions were also observed in ‘World’ and ‘Japan’ than ‘Primitive’ at 10–20 Mb on Chr05, 20–30 Mb on Chr10, and 10–20 Mb on Chr12; suggesting the possibility of selective sweeps during domestication or the breeding process. Furthermore, we focused on somewhat higher polymorphic regions, which are remarkable to the ‘Japan’ subgroup. The region at 4.5 Mb on Chr03, reported as the *Rps1* region, includes the coiled-coil nucleotide-binding site leucine-rich repeat (CC-NBS–LRR)-type gene clusters for Phytophthora resistance.^82^ The 30 Mb region in Chr13, reported as the *Rsv1* region, includes the NBS-LRR resistance gene cluster for the soybean mosaic virus.^83^ Furthermore, the 47 Mb region in Chr14, the *Rsv3* gene region, includes the CC-NBS–LRR gene cluster for the soybean mosaic virus.^84^ It is consistent that these three regions include *R* gene clusters and higher amounts of SVs, and it is thought that genes in such SV rich regions may generate unique alleles in the soybean accessions of the ‘Japan’ subgroup.

### 3.5. Identification of known variations related to the I locus and loci related to colour traits

We further examined the relationship between SVs and seed coat colour phenotypes by comparing known variants related to the chalcone synthase (*CHS*) gene cluster of the *I* locus on Chr08. It has been reported that duplications or deletions of *CHS* genes influence seed coat pigmentation in Rosids.^48^^,^^50^^,^^85^^,^^86^ In soybean, a chimeric sequence consisting of subtilisin and *CHS1* anti-sense of duplicated *CHS* clusters (*Gm-c1069-6017*), has been suggested to cause *CHS* gene silencing and change the yellow seed coat with pigmented hilum, the ‘so-called’ dominant *i^i^* alleles.^41^ Another chimeric sequence (*GmIRCHS*) consisting of *DnaJ* and inverted *CHS3* genes, causes PTGS of *CHS* genes and changes the yellow seed coat with yellow hilum, the ‘so-called’ dominant *I* allele.^40^ The genomic region of the *i^i^* allele is covered by two ‘Williams 82’ BAC clones, which are BAC77G7-a and BAC56G2^70^, and their sequences are located in Chr08 of Gmax_275_v2.0 and are approximately 18 kb apart from each other; from the position of 8,410,306, where the 3' end of BAC56G2 matched to the position of 8,428,210 where the 3' end of BAC77G7-a complementary matched (Supplementary Fig. S9). Although the gene annotations on BAC56G2 were well conserved on Gmax_275_v2.0, the most important genomic region, including Gm-c1069-6017 and *CHS* clusters A and B related to seed coat pigmentation on BAC77G7-a were not correctly assembled and thus were not identified on Gmax_275_v2.0. Similarly, the genomic region with the *GmIRCHS* sequence for the dominant *I* allele was not identified because of ‘Williams 82’ (the donor of Gmax_275_v2.0) having a different *i^i^* allele. Interestingly, we identified chimeric sequences derived from *Gm-c1069-6017* and *GmIRCHS* in the partially aligned PacBio reads on another copy of subtilisin (Glyma.08G109000) and *DnaJ* (Glyma.08G109700), respectively (Supplementary Fig. S9). Furthermore, the short chimeric sequences in the partially aligned Illumina reads near the SV breakpoints were manually recorded as presence and absence variation in all accessions (Supplementary Table S5). The seed coat colour of 53 accessions without both chimeric sequences in the mini-core collections revealed brown, reddish-brown, and black, while the remaining yellow and green seed coat colour accessions had either of the chimeric sequences. Among accessions with yellow and green seed coat colours, no pigmentation was observed on the hilum of 22 accessions with chimeric *GmIRCHS* sequences, while 122 accessions with chimeric partial *Gm-c1069-6017* sequence revealed pigmentation on the hilum. Thus, the PAVs related to the *I* locus in the partially aligned reads successfully explained the variation in seed coat colour and pigmentation on the hilum of 197 accessions.

Among the classical loci that governed the seed coat colour of soybean, we identified new functional alleles at the *R* and *K1* loci based on the read mapping data. Three known non-functional alleles, Gly63fs, Arg75fs, and splice site change (AGgt>AGtt) at the R2R3 MYB gene Glyma.09G235100, which is classically called *R* locus,^42^ distinguished 12 brown seed coat colour accessions from 35 black seed coat colours and two reddish-brown accessions (Supplementary Table S5). Two brown seed colour accessions, GmWMC138 and GmWMC159, were found to have two new non-functional alleles, Glu67del and Asn213fs, respectively. The novel missense variant Trp32Ser was identified as GmWMC019 and GmWMC042. There was no causal variation between black seed accession (GmJMC055) and reddish-brown seed accession (GmJMC099). The reddish-brown colour, however, is somewhat different from the typical brown colour and may be controlled by other genes.

Twenty-six green seed coat colour accessions could be further distinguished from 117 yellow seed coat colour accessions using a known functional stop-loss variant at the CAAX amino-terminal protease gene Glyma.01G198500, which is classically called the *G* locus.^38^ There was no causal variation at Glyma.01G198500 to explain the green seed colour of the GmWMC011 accession. Saddle-shaped black pigmentation on the seed coat of GmJMC003 and GmJMC102 could be explained by non-functional Val351fs allele of the *Argonaute5* gene Glyma.11G190900, which is classically called the *K1* locus.^87^ A new non-functional Leu752stop allele identified in the present study explained the saddle-shaped pigmentation of GmWMC073.

The classical locus *T* encoding flavonoid 3′-hydroxylase (F3′H) gene is known to control pigmentation in the hilum and pubescent.^43^ Among 122 accessions with the *i^i^* allele genetic background, the hilum colour of 76 accessions with functional alleles revealed a brown to black colour, whereas 38 accessions with the non-functional allele Lys389fs had a light brown to brown hilum colour. Three new non-functional alleles, Ile325fs, Ala9Thr+Lys389fs, and Asn322fs+Ile325fs were identified; however, Asn322fs+Ile325fs in GmWMC129 and GmWMC134, and Ile325fs in GmWMC153 did not lighten the hilum colour compared to the other non-functional alleles. These non-functional variants explained the grey pubescence colour of 64 accessions, except for Asn322fs+Ile325fs, where a secondary mutation at 325 bp shifted the offset sequence to the reading frame. The *W1* locus is known to control the colours of the flower and hypocotyl and encode the flavonoid 3′5′‐hydroxylase (F3′5′H) gene.^44^ The known stop-loss variant in the third exon caused by a 53 bp deletion at Glyma.13G072100 was perfectly identified as the phenotype of 142 accessions with a purple flower and hypocotyl.

The stay-green phenotype in the mini-core collections was characterized by the colour of the cotyledon and leaf at maturity. Among the 13 accessions with the stay-green phenotype, nine accessions were found to have heterozygous variant Ile25fs caused by a 5-bp insertion, which led to a frameshift in the *psbM* gene Glyma.15G208300. Stay-green controlled by *cytG* is known to be the same 5-bp insertion on chloroplast *psbM*, which encodes small subunits of photosystem II.^36^ Since organelle genome sequences did not include read mapping in the present study, the reads including chloroplast *psbM* have been mapped to the nuclear *psbM* gene Glyma.15G208300 and were detected as heterozygous variant Ile25fs. The remaining stay-green accessions were characterized as having double recessive genes, *d1* and *d2*.^35^ The *D1* and *D2* loci encode the *GmSGR1* gene Glyma.11G027400 and *GmSGR2* gene Glyma.01G214600, respectively. For the *D2* locus, the non-functional known variant, Val60fs, and the new non-functional variant, Lys59fs, at *GmSGR2* gene Glyma.01G214600 were observed at five and three accessions, respectively. In contrast, a non-functional known variant of the *D1* locus by GmD2IN transposon insertion^35^ to *GmSGR1* gene Glyma.11G027400 was identified from five accessions by manual inspection of the partially aligned Illumina reads to the corresponding genomic region (Chr11:1975880 or Chr11:1975350). Among them, only four accessions (GmWMC011, GmWMC018, GmWMC127, and GmWMC129) revealed a stay-green phenotype by the non-functional variant at both loci.

### 3.6. Survey of rearrangement between soybean genomes during progression based on CNV analysis

As investigations related to the diversity of soybean accessions advanced, we performed CNV analysis for the 198 accessions with Illumina reads, which indicated the trace of the genomic rearrangements (Supplementary Fig. S10).^13^^,^^14^ The distributions of CNVs were generally similar in the three subgroups, indicating that there were only a few large-scale genome rearrangements characterizing subgroups, unlike sequence variances such as SNPs and indels. However, such rearrangements were also observed in some specific accessions such as ‘Bongchubalejama’ (GmWMC089) in 13–16 Mb of Chr01 in the ‘Japan’ subgroup. We confirmed read mapping of these regions, which included long gaps of more than 1 Mb with extremely low read coverage, and found that many genes in these regions have been lost. Accordingly, the phenotype of such accessions with a long gap would be influenced if the target gene of interest lacks in the region. A similar long gap was found in ‘DAIZU’ (GmJMC133) at 30.2–31.0 Mb on Chr08 of the ‘Japan’ subgroup; ‘HOUJAKU’ (GmJMC067) at 14.9–15.7 Mb on Chr10 of the ‘Japan’ subgroup; approximately 24–28 Mb on Chr12 of ‘MEGURO 1’ (GmJMC064) and ‘POCHAL’ (GmWMC020) of the ‘Japan’ subgroup and ‘GREEN PE POKE’ (GmWMC127) and ‘IPPON SANGOU’ (GmJMC076) of the ‘World’ subgroup. This CNV information will contribute to association analysis and gene functional analysis.

## 4. Conclusions

In the present study, we obtained the whole-genome sequences of 198 soybean accessions, which had been carefully selected to represent the genetic diversity of the worldwide 1,603 accessions^31^ and particularly represented the variations in Japanese accessions harbouring the distinct genetic and morphological characteristics from those in the Asian continent.^32^^,^^33^ The genetic diversity of the 198 accessions was analysed based on their whole-genome sequences, and then the existence of three subgroups that contain specific and shared polymorphisms was suggested. GWAS on DTF and the detailed comparison between sequence polymorphisms and phenotypes in colour-related traits were performed with the whole-genome sequences of the accessions. The variations in DTF and colour related to the seed coat, hilum, pubescence, and flower were mostly characterized by using the known and new variants observed in the whole-genome sequence reads. CNV analysis suggested that only a few large-scale genome rearrangements may have occurred during the domestication of soybeans. The results suggested that the genomic sequences and variants obtained for the 198 soybean accessions have great potential to provide information for soybean breeding and genetic studies to uncover novel alleles or genes involved in agronomically important traits.

## Supplementary data

Supplementary data are available at *DNARES* online.

## Supplementary Material

dsaa032_Supplementary_Data
