# Supplementary material for: Whole-genome sequence diversity and association analysis of 198 soybean accessions in mini-core collections
Source: DNA Res. 2021 Jan 25;28(1):dsaa032. doi: 10.1093/dnares/dsaa032 (PMC7934572; doi:10.1093/dnares/dsaa032)
Supplement: dsaa032_Supplementary_Data [file dsaa032_supplementary_data.zip › Supplementary Methods.docx]

**Supplementary Methods**

Variant data preparation

The ‘initial-step’ variant dataset (dataset 1) contained 10,116,707 SNPs and 2,835,680 indels. We derived three separate datasets from the initial dataset 1. After removing the indels from dataset 1, the set of 10,116,707 SNPs was used as dataset 2. For datasets 3 and 4, we replaced heterozygous genotypes with missing data based on the assumption that all markers were fixed in all the accessions in the mini-core collections. The minor allele frequency (MAF) ≥ 0.025 was set as dataset 3 as an additional filter. Imputation with Beagle 5.0 was conducted on both datasets using default parameter settings 1. Dataset 4 was selected with an LD-pruning method to remove the effects of high LD SNPs. LD calculation was performed using the PLINK 1.9^2^ on a subset of selected markers by independent pairwise LD pruning: window size at 50, step size at 5, and *r^2^* at 0.5. Markers showing higher linkage disequilibrium (LD) values greater than *r^2^* = 0.5 were removed according to the LD measured as squared Pearson correlation coefficient 11.

Dataset 1 was used for variant analysis with SnpEff^3^ based on the Annotation Information (Gmax_275_Wm82.a2.v1.gene.gff3 from Phytozome v12.1)^4^ with default parameters. The annotated variants were visualized with the gene annotations using the multiple genome browser TASUKE version 1.5.^5^ Dataset 2, consisting of 10,116,707 SNPs, was used to estimate nucleotide diversity, population differentiation, and linkage disequilibrium. Dataset 3, consisting of 4,776,813 SNPs, was used in GWAS. Dataset 4, consisting of 1,792,931 SNPs, was used to construct a phylogenetic tree, ADMIXTURE^6^ clustering, and principal component analysis (PCA).

1. Browning, B. L., Zhou, Y., and Browning, S. R. 2018, A one-penny imputed genome from next-generation reference panels, *Am. J. Hum. Genet*., **103**, 338–348.

2. Chang, C. C., Chow, C. C., Tellier, L. C., Vattikuti, S., Purcell, S. M., and Lee, J. J. 2015, Second-generation PLINK: rising to the challenge of larger and richer datasets, *Gigascience*, **4**, 7.

3. Cingolani, P., Platts, A., Wang, L. L., et al. 2012, A program for annotating and predicting the effects of single nucleotide polymorphisms, SnpEff. Fly, **6**, 80–92.

4. Goodstein, D. M., Shu, S., Howson, R., et al. 2012, Phytozome: a comparative platform for green plant genomics. *Nucleic Acids Res*., 40, D1178–D1186.

5. Kumagai, M., Kim, J., Itoh, R., and Itoh, T. 2013, TASUKE: a web-based visualization program for large-scale resequencing data, *Bioinformatics*, **29**, 1806–1808.

6. Alexander, D. H., Novembre, J., and Lange, K. 2009, Fast model-based estimation of ancestry in unrelated individuals, *Genome Res*., **19**, 1655–1664.
