# Supplementary material for: Whole-genome sequence diversity and association analysis of 198 soybean accessions in mini-core collections
Source: DNA Res. 2021 Jan 25;28(1):dsaa032. doi: 10.1093/dnares/dsaa032 (PMC7934572; doi:10.1093/dnares/dsaa032)
Supplement: dsaa032_Supplementary_Data [file dsaa032_supplementary_data.zip › Supplementary_Fig.ver.3.pdf]

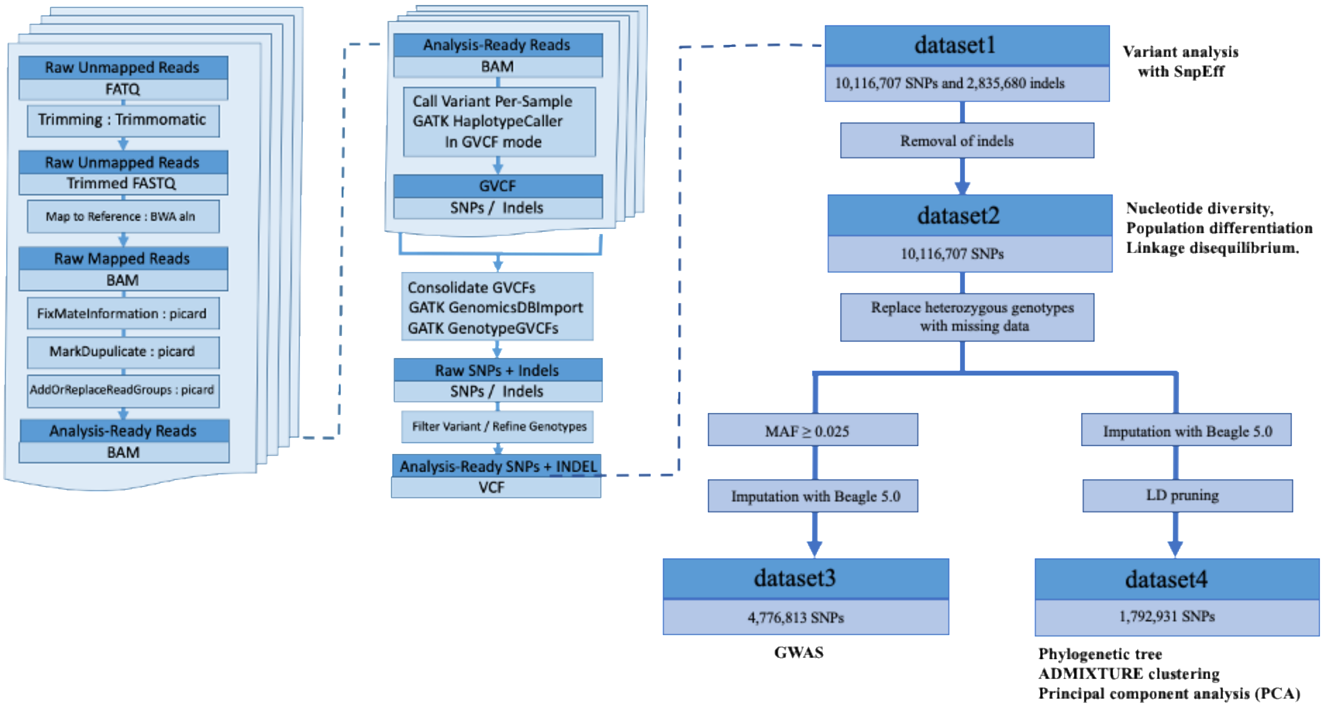

Supplementary Fig. S1. Variant data preparation pipeline.

(A)

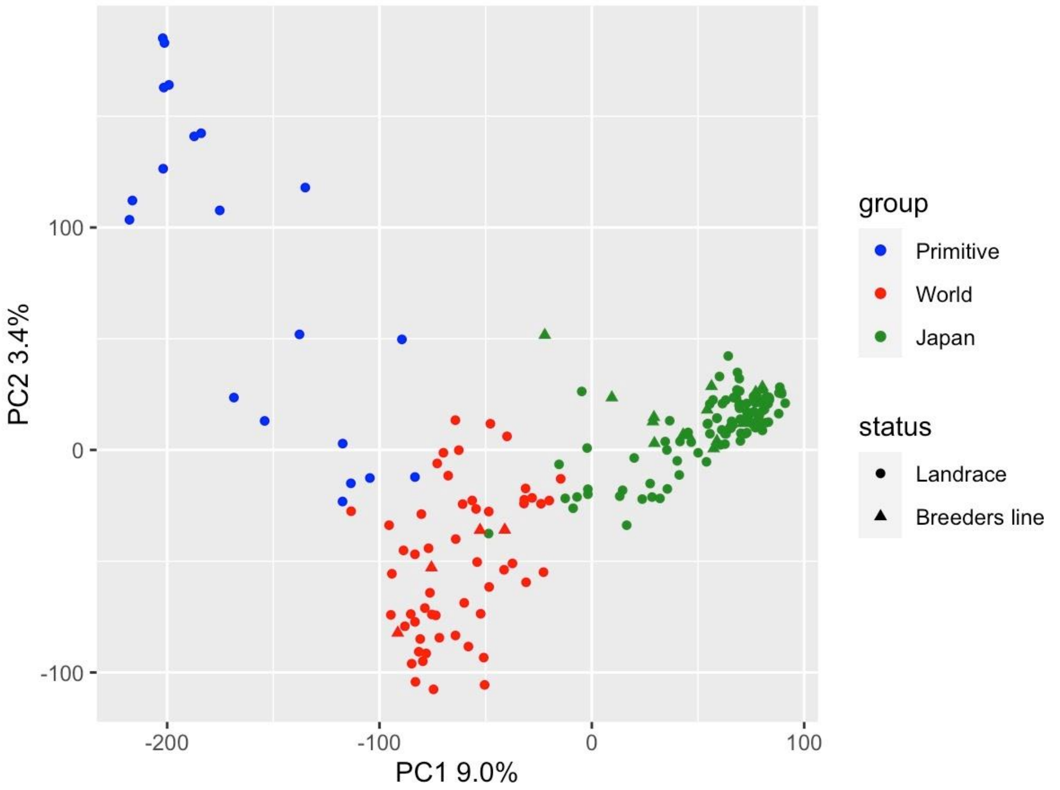

(B)

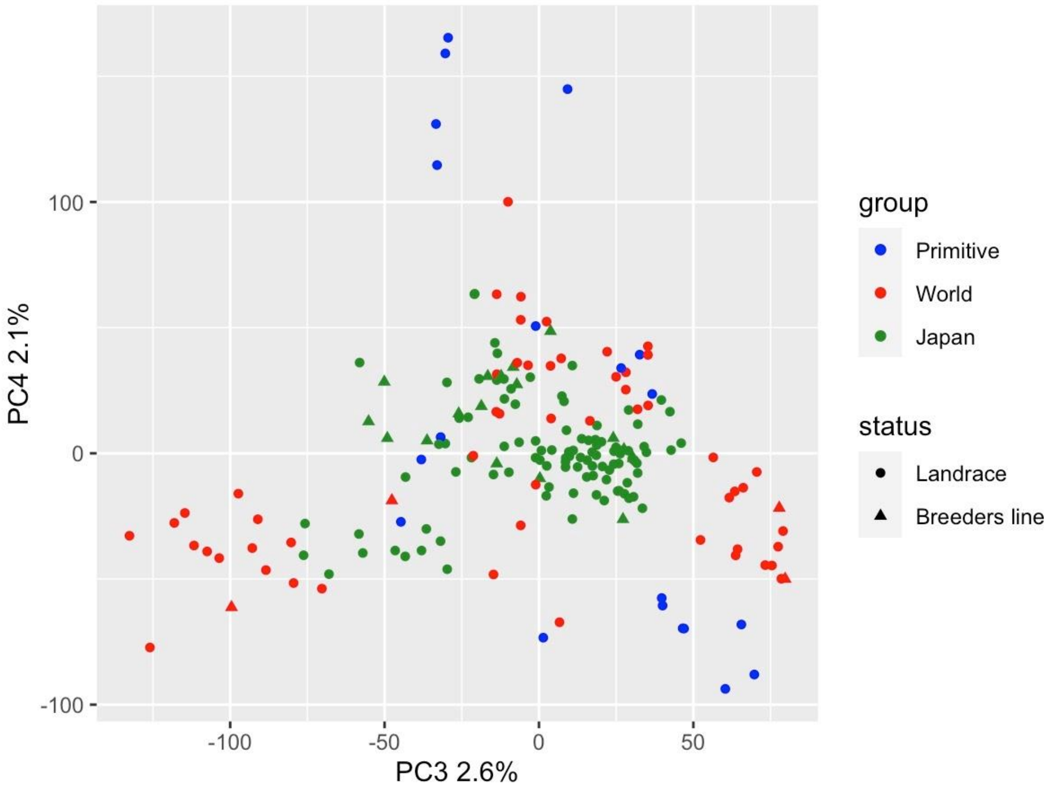

Supplementary Fig. S2. The Scatter plots of principal component (PC) analysis of genome-wide SNP genotypes. (A) 1st and 2nd components (PC1 and PC2), (B) 3rd and 4th components (PC3 and PC4).

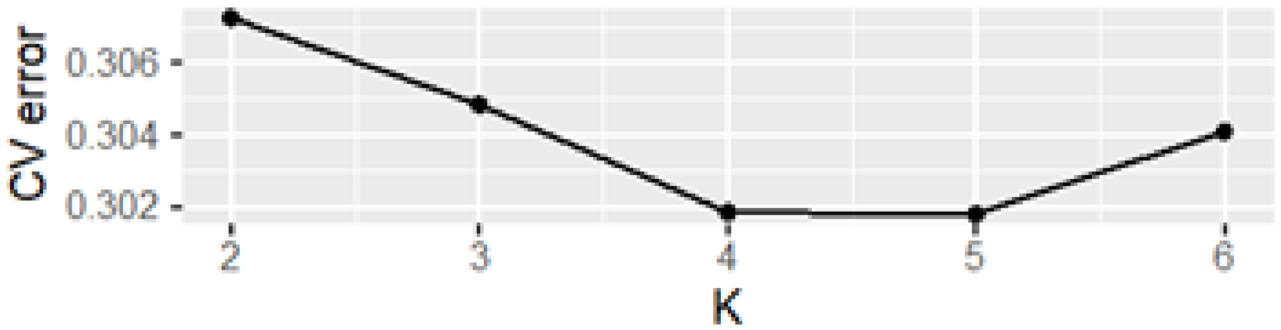

Supplementary Fig. S3. Cross-validation (CV) errors returned by ADMIXTURE.

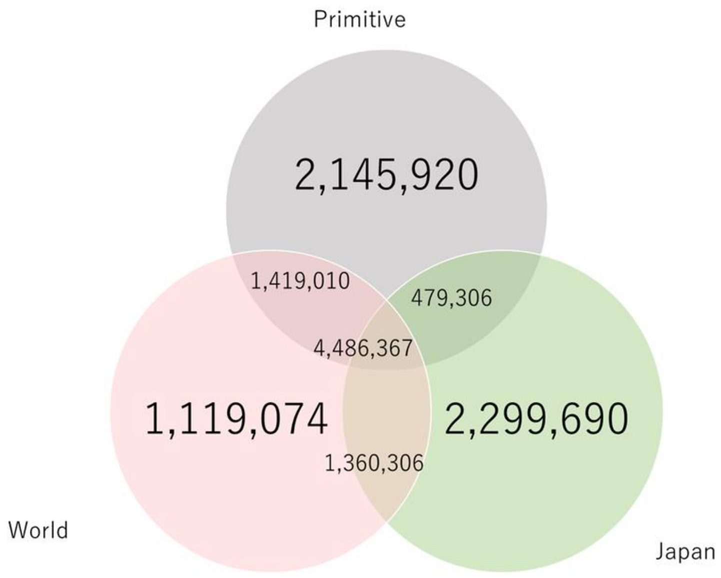

Supplementary Fig. S4. Number of shared variants representing population divergence.

Number of shared variants among Primitive, World and Japan groups.

(A)

(B)

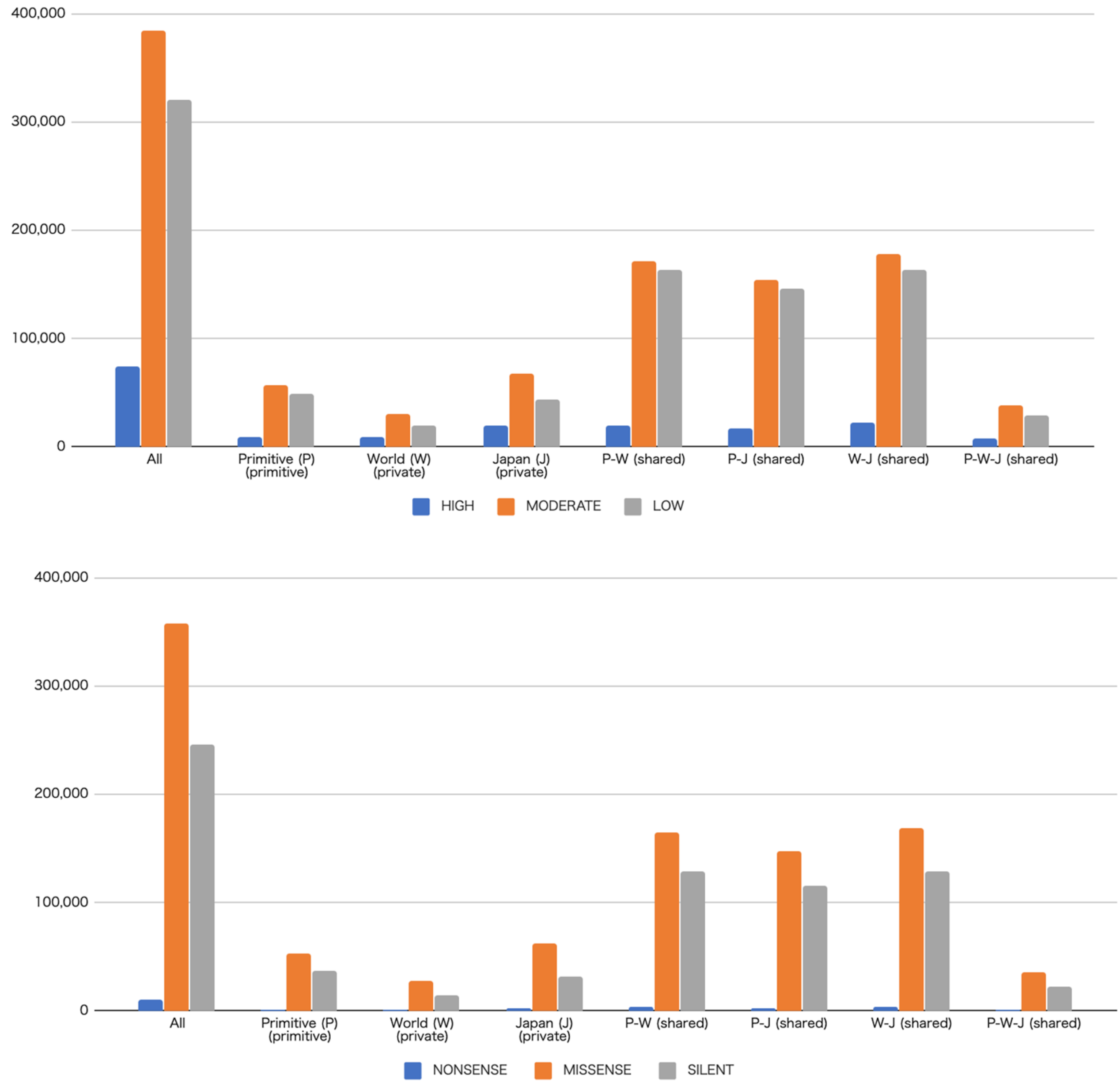

Supplementary Fig.S5. The functional impacts and the functional classes of variants for private variants in "Primitive", "World", and "Japan", variants shared with the pairs of "Primitive" and "World" (P-W), "Primitive" and "Japan" (P-J), "World" and "Japan", and variants shared by all subgroups.

(A) Effects are categorized by 'impact' (HIGH, MODERATE, LOW).

Of 777,282 variants classified into "High", "Moderate" and "Low", 131,698 (17%) and 115,085 (15%) were the private variants of "Japan" and "Primitive", respectively, while 58,935 (8%) were the private variants of "World" (Table S3). The number of the private variants classified into "Moderate" was also the largest (67,804) in "Japan" among the three subgroups (i.e., 56,336 in "Private" and 30,421 in "World"). The number of variants shared by two subgroups did not show clear differences among the pairs of the subgroups; "Primitive-World (P-W)", "Primitive-Japan (P-J), and "World-Japan (W-J)"

(B) Effects are categorized by 'functional class' (NONSENSE, MISSENSE, SILENT).

The number of the private variants classified into "Nonsense" was the largest (2,382) in Japan among the three subgroups (1,476 in "Primitive" and 1,187 in "World"). The number of the private variants classified into "Missense" is also the largest (62,178) in "Japan" among the three groups (52,872 in "Primitive" and 27,845 in "World"). As same as the functional impact, the number of variants shared by two subgroups did not show a clear difference among the pairs of the subgroups (Fig. S2B). Of 30,432,585, the greatest number of variants were located in intergenic regions (11,800,945; 39%),, followed by upstream (7,657,111; 25%) and downstream (6,911,085; 23%) regions of genes. Also, 2,765, 842 (9%) of the variants are in the intron and 691,617 (2%) in the exon (Table S3).

(A)

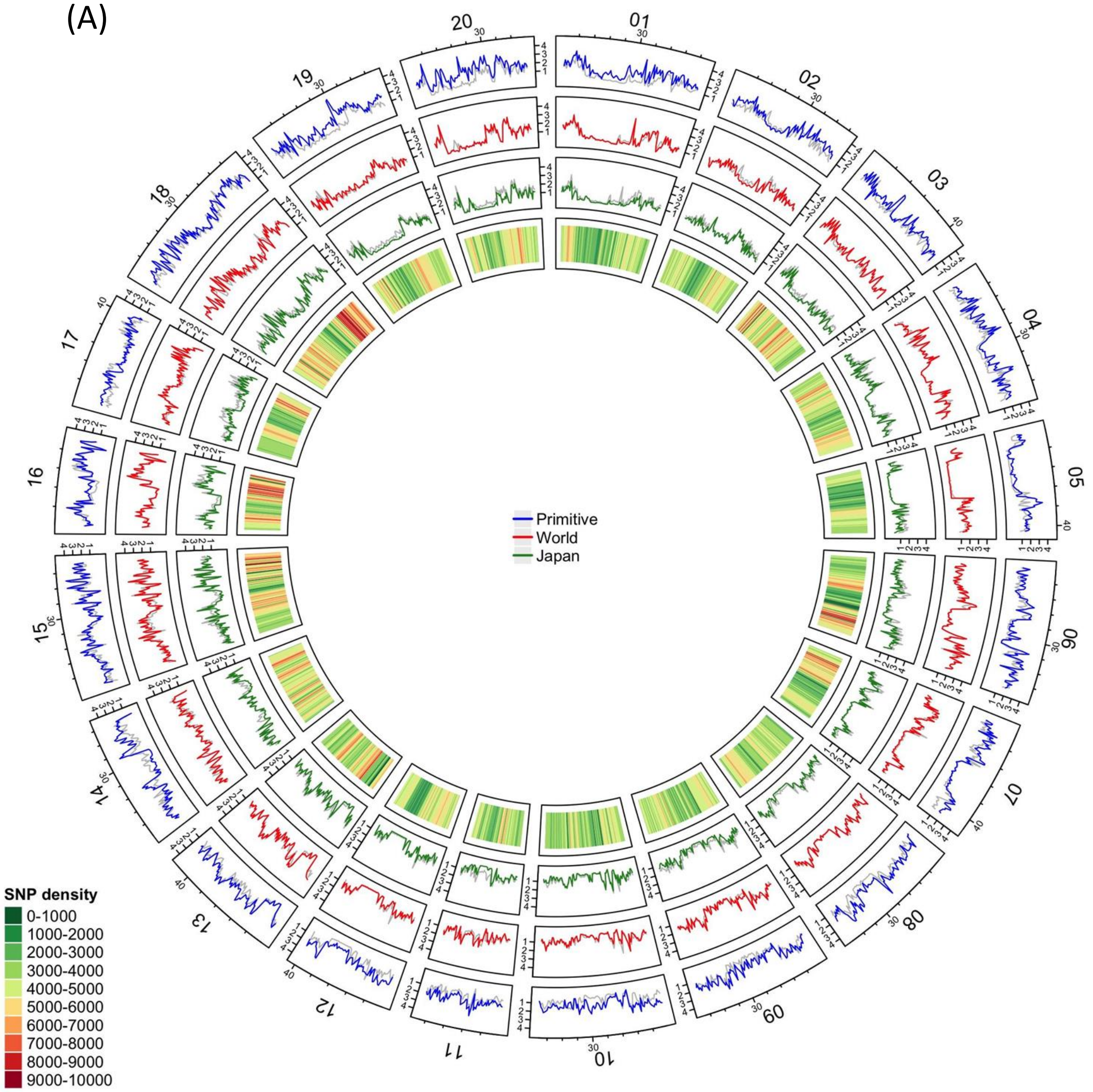

Supplementary Fig.S6A. Genome-wide pattern of Nucleotide diversity (π) .
The gray lines indicate the values calculated from all data together.

(B)

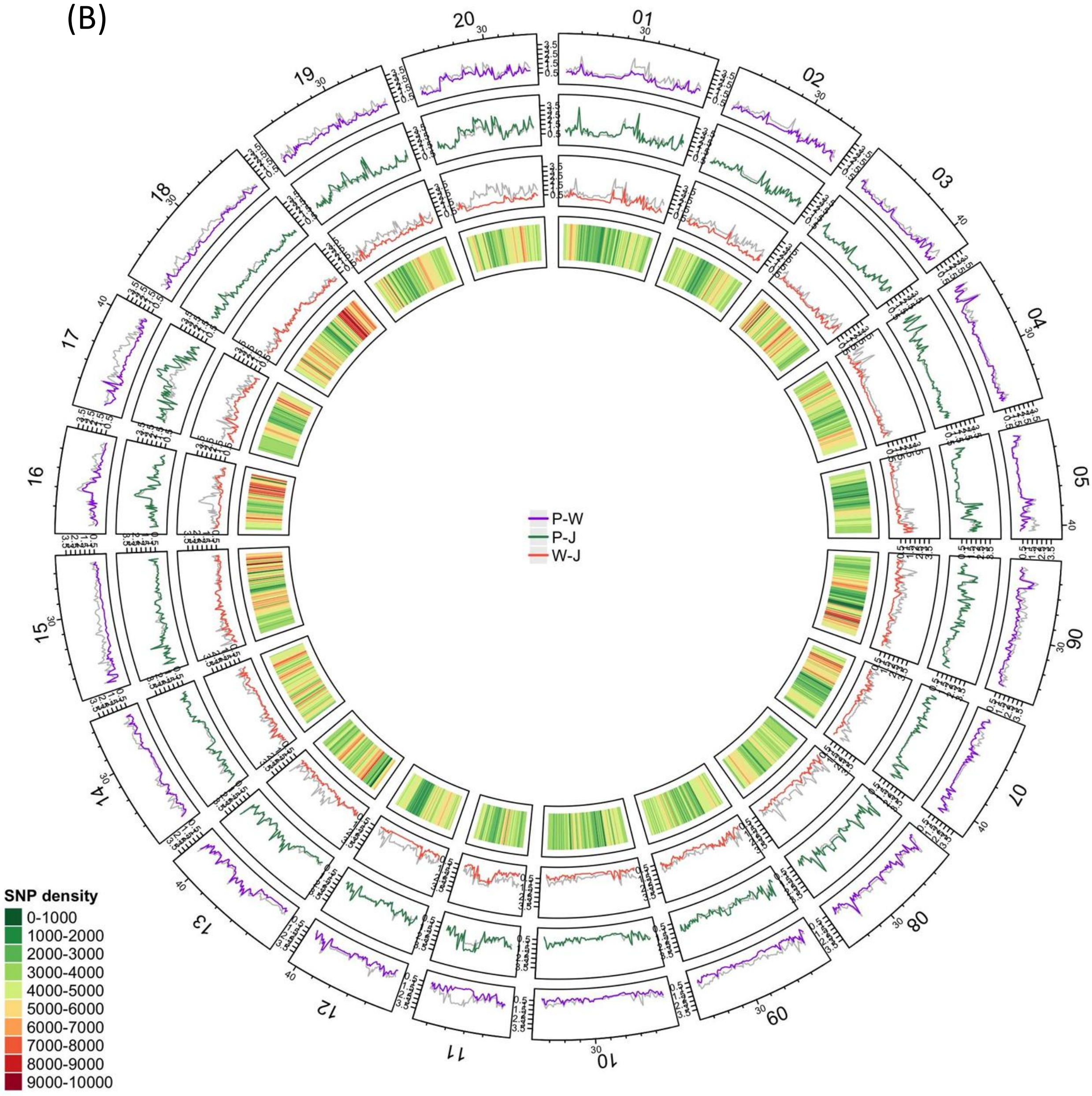

Supplementary Fig.S6B. Genome-wide pattern of the genetic differentiation between subpopulations.

The gray lines indicate the values calculated from all data together.

(C)

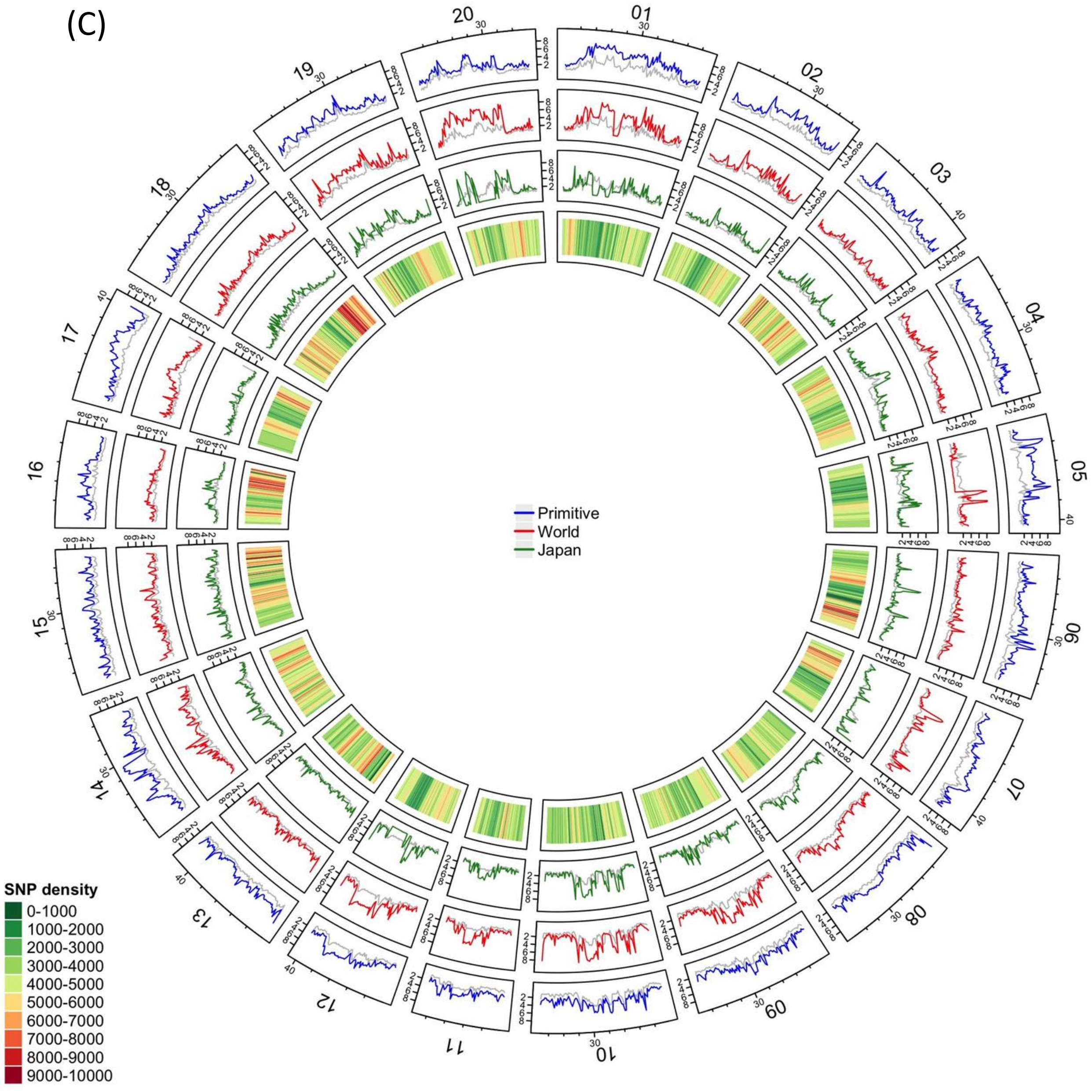

Supplementary Fig.S6C. Genome-wide pattern of the linkage disequilibrium. The gray lines indicate the values calculated from all data together.

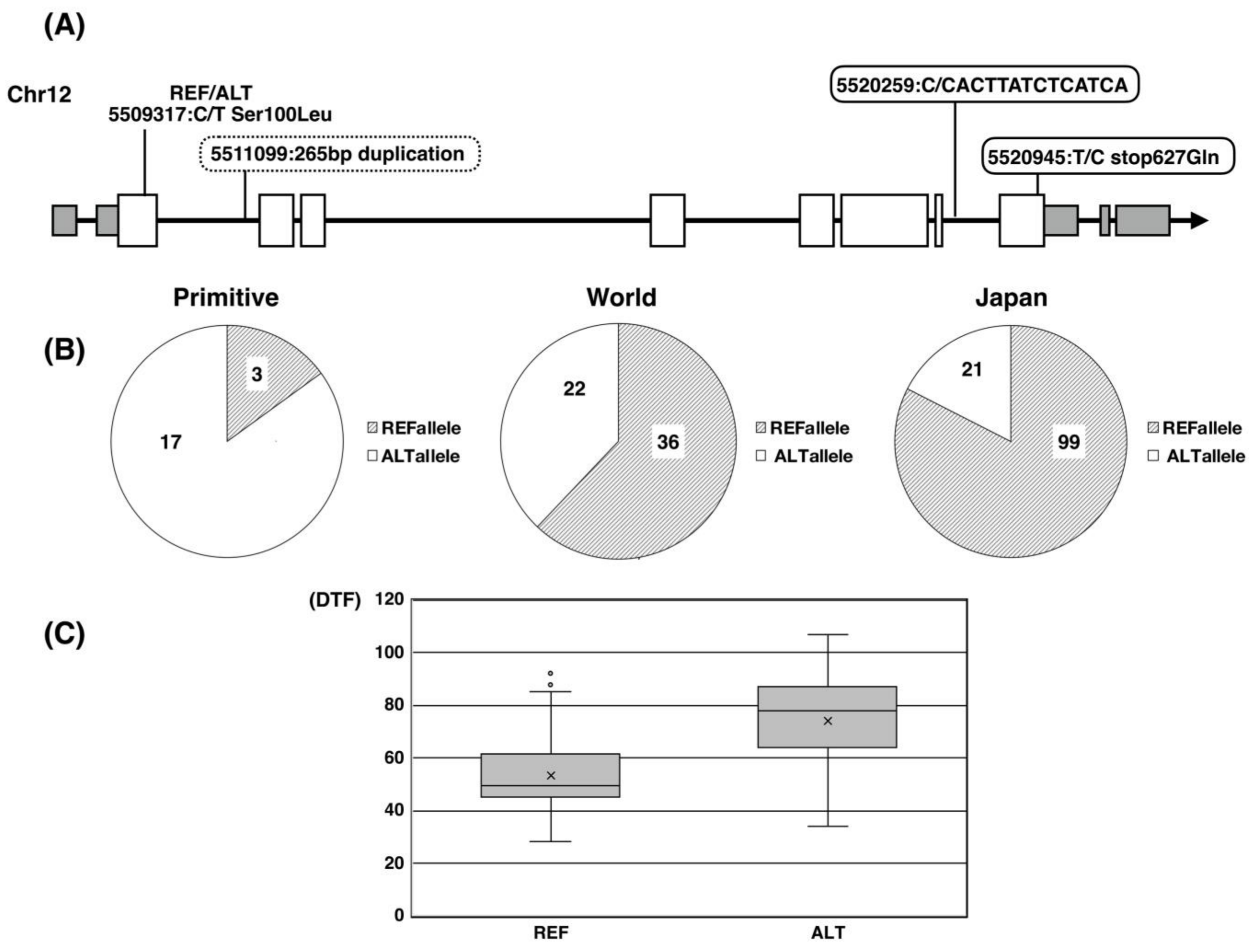

Supplementary Fig. S7. Sequence variants on GmPRR3b gene (Glyma.12G073900) with two-component response regulator-like APRR3 genes, allele frequency of a targeted SNP, and distribution of flowering date.

(A) The structures of Glyma.12G073900 and identified variants. Black arrow, white and gray box represent gene direction, coding exon and UTR, respectively. Genomic positions represent variant identified by Illumina reads in the 198 soybean accessions. Those enclosed with solid and dot lines were basic variants used in GWAS and SVs detected by PacBio, respectively.

(B) Allele frequency in Primitive, World, and Japan groups of the 198 accessions of the SNP (Ch12-5520945) that showed the highest significance on the gene by the GWAS analysis. Ref (same as the reference sequence, Williams 82) and Alt alleles are "T" and "C", respectively. The numbers in the pie charts indicate that of accessions having the alleles.

(C) Boxplots of flowering date in the 184 accessions having Ref (left) and Alt (right) allele on the SNP (Chr12, 5520945). The vertical line indicates the days to flowering (DTF). The vertical line indicates the days to flowering (DTF).

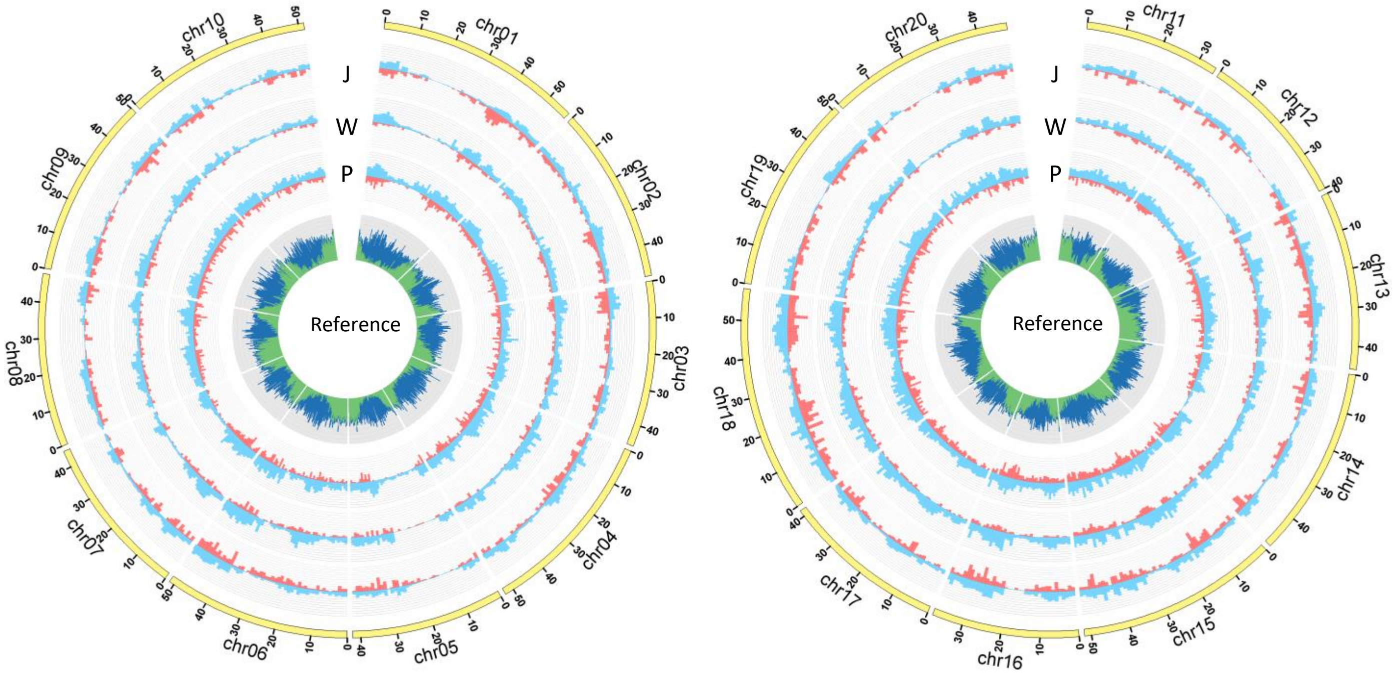

Supplementary Fig. S8. Distribution of average SVs and pi values in Japan, Wild and Primitive groups of the 10 PacBio sequenced lines.

'Reference' shows Ratios of gene (green) and transposon (blue) in 1 Mb window in the Williams 82 genome. P, W and J represent averages of SV numbers (red) and π values (aqua) in primitive, world and, respectively. The averages of SV numbers and pi values were calculated in 1Mb window. The distance between horizontal lines are 2.5 and 0.001, respectively.

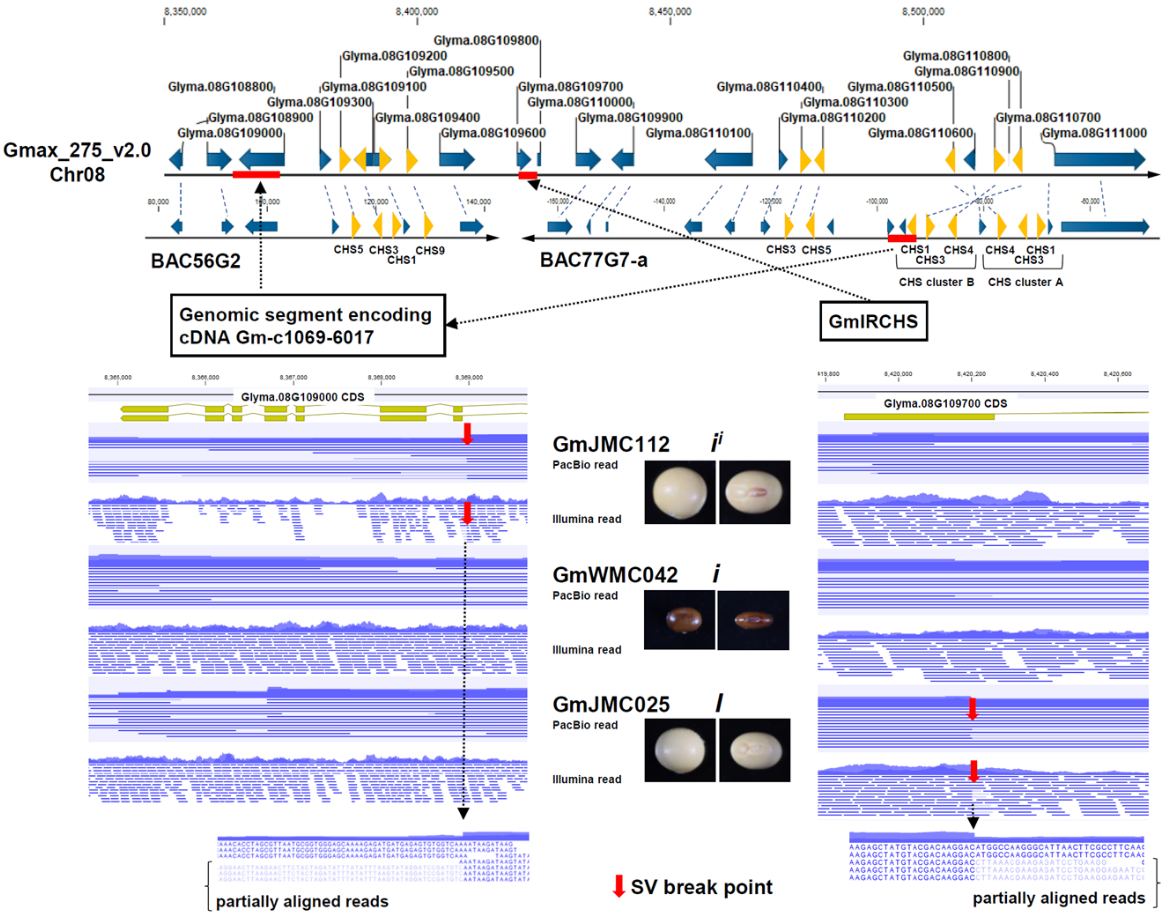

Supplementary Fig. S9. Presence and absence of the chalcone synthase (CHS) genes in the clusters on BAC56G2 and BAC77G7-a of BAC sequence, which cover soybean I locus.

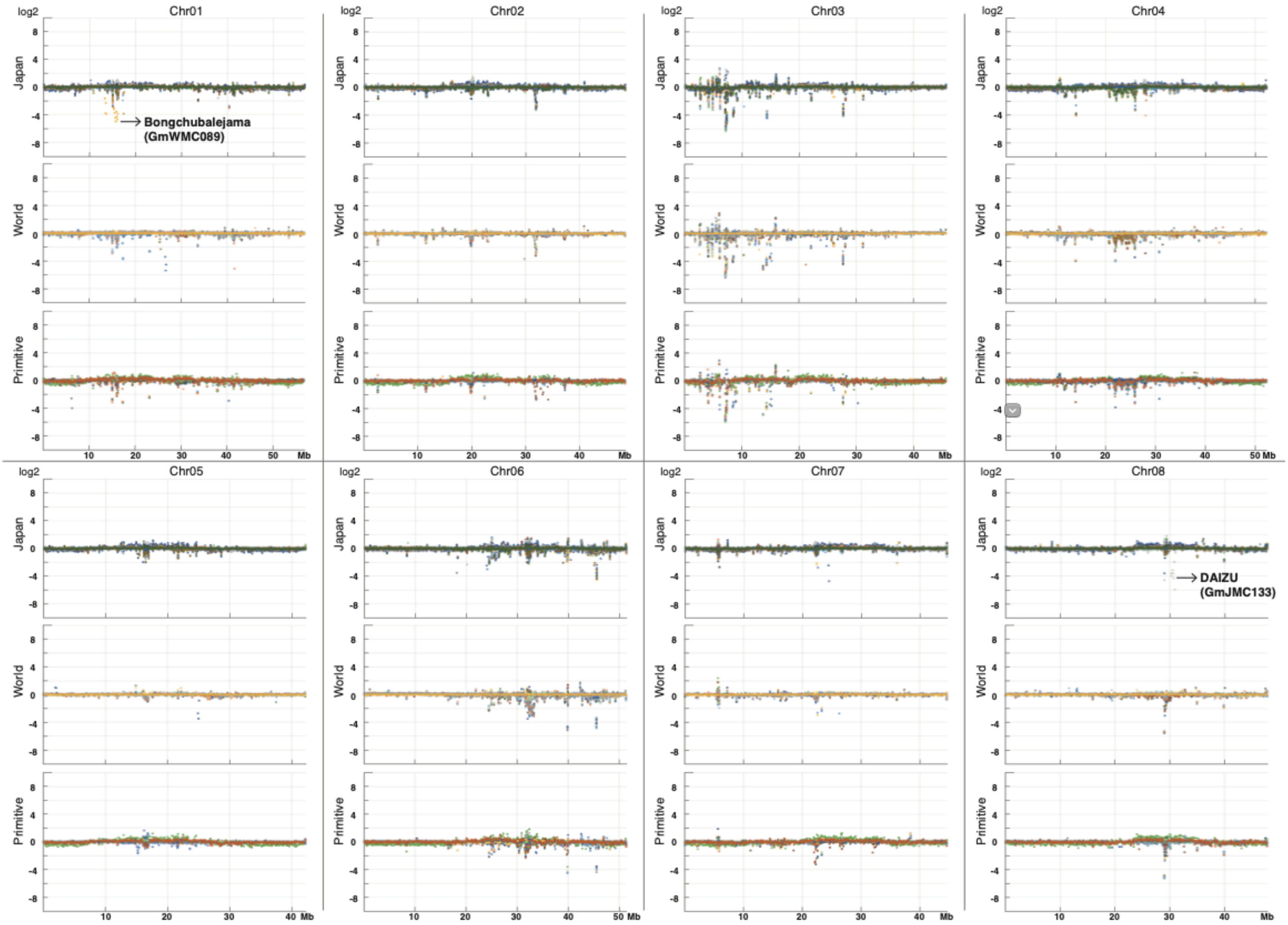

Supplementary Fig. S10. CNV analysis of soybean mini core collection.
The plots of the accessions were represented separately for chromosomes and their groups.
The horizontal of the graphs indicate chromosome positions and the vartical indicate log2 ratios. Respective accession is represented by different plot color. The details are illustrated in the last of this figure.

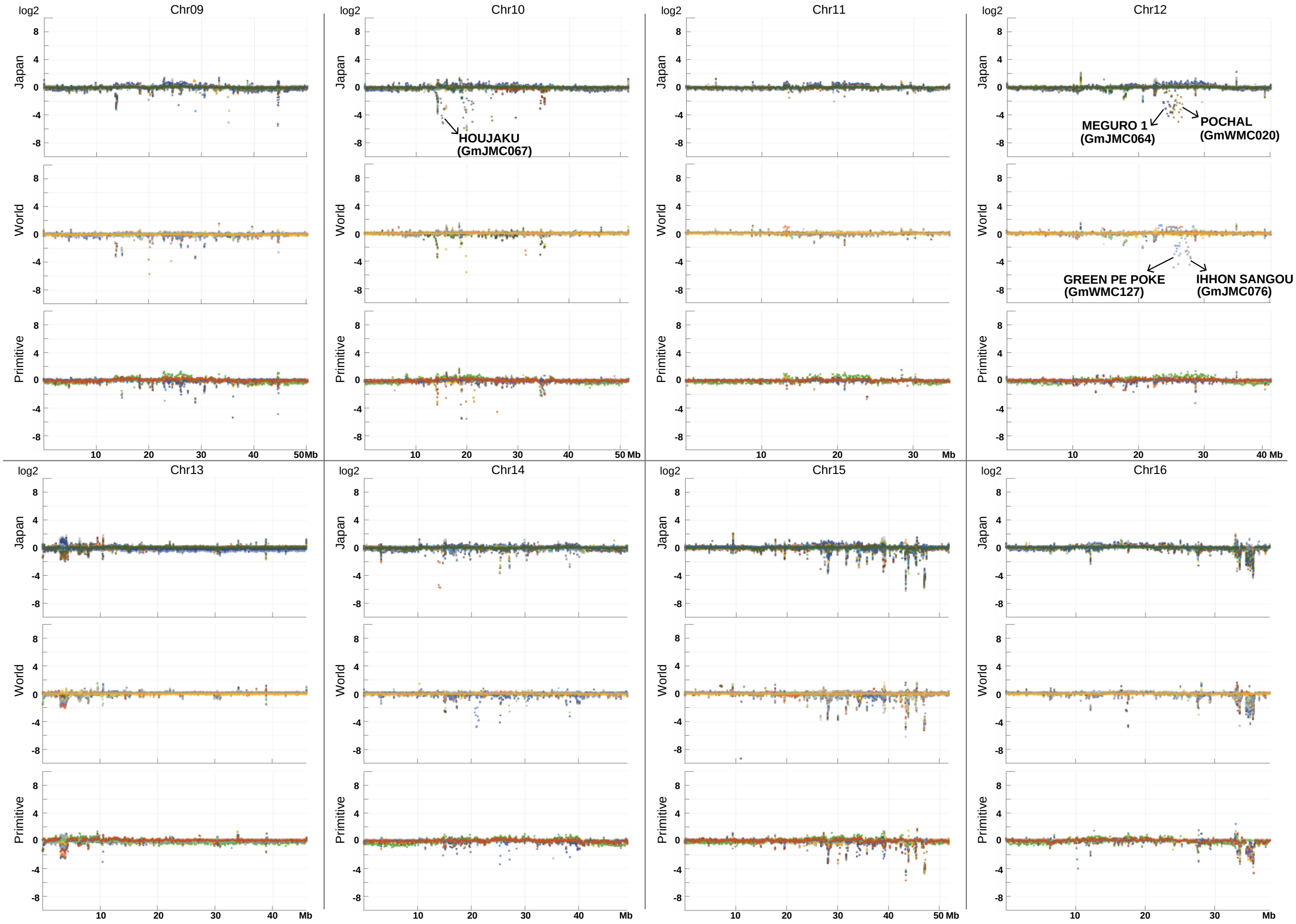
Chr09
Chr10
Chr11
Chr12
Chr13
Chr14
Chr15
Chr16
log2
Japan
World
Primitive
HOUJAKU
(GmJMC067)
MEGURO 1
(GmJMC064)
POCHAL
(GmWMC020)
GREEN PE POKE
(GmWMC127)
IHHON SANGOU
(GmJMC076)
Mb

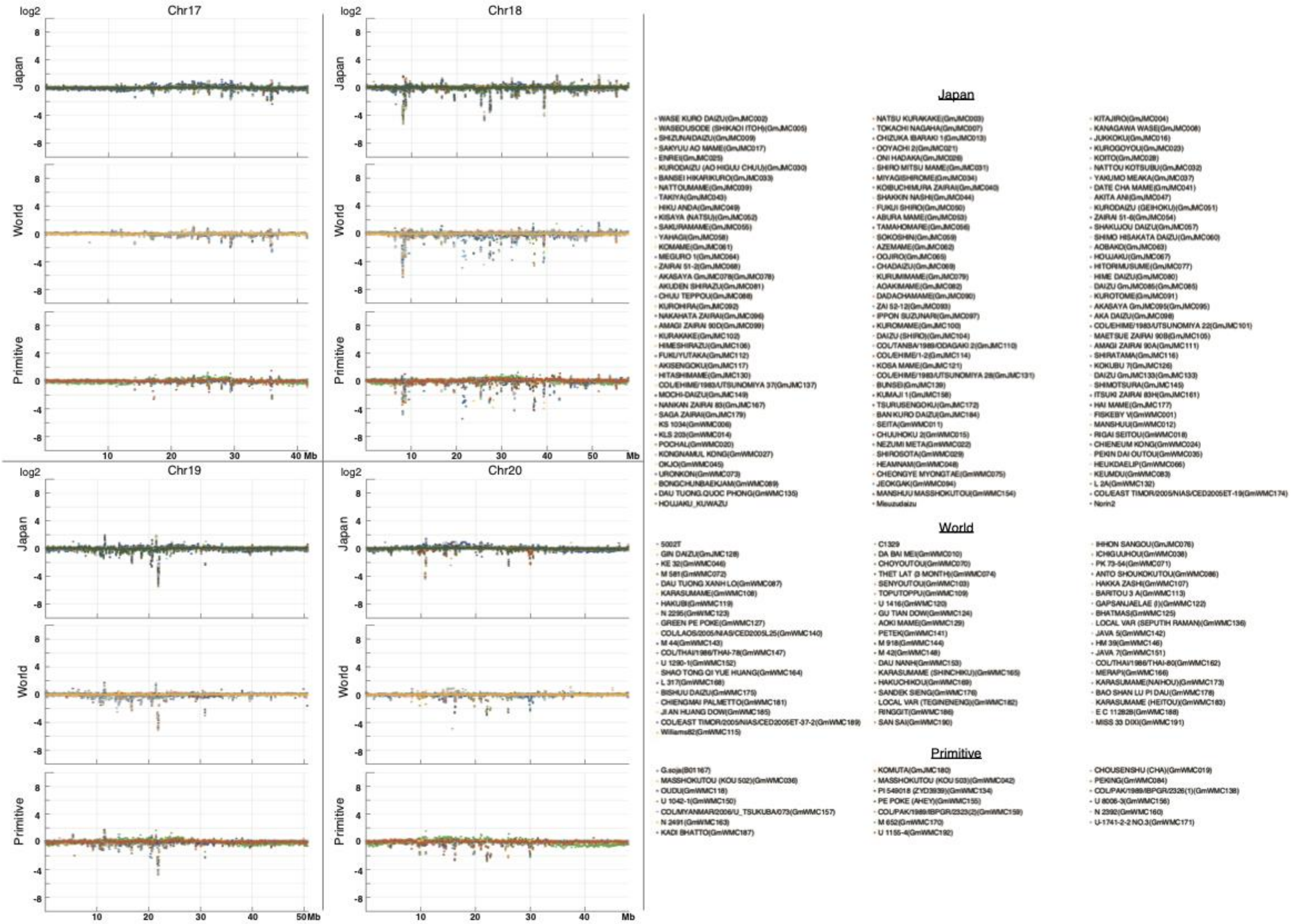

Chr17
Chr18
Chr19
Chr20
log2
Japan
World
Primitive
Mb
Japan
World
Primitive
